# Genome diversity and highland-adaptative variation in Tibet barley landrace population of China

**DOI:** 10.3389/fpls.2023.1189642

**Published:** 2023-05-10

**Authors:** Dawa Dondup, Yang Yang, Dongdong Xu, Lhundrup Namgyal, Zihao Wang, Xia Shen, Tsechoe Dorji, Nyima kyi, Lhakpa Drolma, Liyun Gao, Zhuo Ga, Zha Sang, Zhuo Ga, Wang Mu, Pubu Zhuoma, Xiongnu Taba, Guocheng Jiao, Wenhua Liao, Yawei Tang, Xingquan Zeng, Zhaxi Luobu, Yufeng Wu, Chunchao Wang, Jing Zhang, Zengjun Qi, Weilong Guo, Ganggang Guo

**Affiliations:** ^1^ State Key Laboratory of Hulless Barley and Yak Germplasm Resources and Genetic Improvement, Research Institute of Agriculture, Tibet Academy of Agriculture and Animal Husbandry Sciences, Lhasa, China; ^2^ College of Plant Science, Tibet Agricultural and Husbandry University, Linzhi, China; ^3^ College of Life Sciences, Zaozhuang University, Zaozhuang, China; ^4^ Key Laboratory of Grain Crop Genetic Resources Evaluation and Utilization (MARA), The National Key Facility for Crop Gene Resources and Genetic Improvement, Institute of Crop Sciences, Chinese Academy of Agricultural Sciences (ICS-CAAS), Beijing, China; ^5^ Institute of Industrial Crops, Shandong Academy of Agricultural Sciences, Jinan, China; ^6^ Frontiers Science Center for Molecular Design Breeding, China Agricultural University, Beijing, China; ^7^ State Key Laboratory of Crop Genetics and Germplasm Enhancement, Nanjing Agricultural University, Nanjing, China; ^8^ Key Laboratory of Alpine Ecology and Biodiversity, Institute of Tibetan Plateau Research, Chinese Academy of Sciences, Beijing, China; ^9^ Center for Excellence in Tibetan Plateau Earth Science, Chinese Academy of Sciences, Beijing, China; ^10^ Tibet Climate Center, Tibet Meteorological Bureau, Lhasa, China; ^11^ Tibet Institute of Plateau Atmospheric and Environmental Sciences, Tibet Meteorological Bureau, Lhasa, China; ^12^ Key Laboratory of Atmospheric Environment of Tibet Autonomous Region, Tibet Meteorological Bureau, Lhasa, China

**Keywords:** highland adaptation, naked barley, tGBS, vernalization genes, grain color

## Abstract

Barley landraces accumulated variation in adapting to extreme highland environments during long-term domestication in Tibet, but little is known about their population structure and genomic selection traces. In this study, tGBS (tunable genotyping by sequencing) sequencing, molecular marker and phenotypic analyses were conducted on 1,308 highland and 58 inland barley landraces in China. The accessions were divided into six sub-populations and clearly distinguished most six-rowed, naked barley accessions (Qingke in Tibet) from inland barley. Genome-wide differentiation was observed in all five sub-populations of Qingke and inland barley accessions. High genetic differentiation in the pericentric regions of chromosomes 2H and 3H contributed to formation of five types of Qingke. Ten haplotypes of the pericentric regions of 2H, 3H, 6H and 7H were further identified as associated with ecological diversification of these sub-populations. There was genetic exchange between eastern and western Qingke but they shared the same progenitor. The identification of 20 inland barley types indicated multiple origins of Qingke in Tibet. The distribution of the five types of Qingke corresponded to specific environments. Two predominant highland-adaptative variations were identified for low temperature tolerance and grain color. Our results provide new insights into the origin, genome differentiation, population structure and highland adaptation in highland barley which will benefit both germplasm enhancement and breeding of naked barley.

## Introduction

The Qinghai-Tibet Plateau is the highest cropping plateau region on earth (Song, 2013). The environment is harsh and considered by some to be unfit for human habitation. On the contrary, the Plateau not only has a sophisticated human culture but also well-adapted plant and animal species. Naked barley (*Hordeum vulgare* L.) called “Qingke” in Chinese and “Ne” in Tibetan is an important food crop in the region. Qingke appeared in written records about 1,300 years ago but was grown in Tibet much earlier. Archaeological evidence shows that wheat and barley that originated in the Middle East were introduced to China about 5,200 years ago, and farmers brought barley to the Tibet Plateau about 3,600 years ago (Zeng et al., 2018; Zhou et al., 2020). Due to its cold tolerance and short growing season, barley adapted to the extreme environment of the Qinghai Tibet Plateau (characterized by cold, drought, strong sunshine, and barren soil) and became the staple food crop and foundation for human settlement in the region (Chen et al., 2015; Guedes et al., 2015; Zhou et al., 2020).

The origin and evolution of Qingke is a fascinating story. Variants with brittle rachis and six-rowed spikes collected from Tibet were named *agriocrithon* (*H. vulgare agriocrithon*) (Åberg, 1938). A large number of *agriocrithon* accessions collected from Tibet display wide genetic diversity, and one theory was that *agriocrithon* is the progenitor of six-rowed barley and originated from Tibet (Shao, 1982; Xu, 1982; Ma, 2000). Considerable genomic research supported this view (Dai et al., 2012; Dai et al., 2014). However, analysis of the allelic variation of *HvBtr1* and *HvBtr2* proved that *agriocrithon* was introduced into Tibet from outside rather than independent origin within the region. Recent archaeological and genomic evidence proved that Qingke was introduced from outside (Chen et al., 2015; Guedes et al., 2015; Pourkheirandish et al., 2015; Pourkheirandish et al., 2018; Zhou et al., 2020).

The Tibet Autonomous Region of China, located in the core of the Qinghai Tibet Plateau, has unique geographic features, deep river valleys in the south and east, surrounded by high mountains in the north, and wide valleys and lake basins in the center (Yang, 1983). Genetic variations related to highland adaptability exist in human, animal and plant populations. For example, the hemoglobin gene *EPAS1* was positively selected in Tibetans and a similar gene segment of ancient Denisovans contributed to adaptation of Tibetan people to highland environments (Simonson et al., 2010; Huerta-Sánchez et al., 2014; Chen et al., 2019). Specific genes for adaptation to hypoxic environments are found in yaks, Tibetan pigs and Tibetan chickens (Qiu et al., 2012; Qiu et al., 2015; Li et al., 2013; Wang et al., 2015). Dramatic contractions and expansions of gene families were likely key mechanisms for *Crucihilaya himalaica* to adapt to highland environments (Zhang et al., 2019). Specific SINE transposons promote adaptation of *Prunus* species to high altitudes by enhancing beneficial metabolites (Wang et al., 2021b).

Altitude, rainfall, light intensity, and high UV radiation of the Tibet plateau contribute to a unique agricultural ecosystem (Yang, 1983; Song, 2013) that led to morphological diversity among Qingke landraces selected under relatively isolated conditions. In order to preserve, protect and make better use of these genetic resources, scientists collected Qingke landraces over more than 30 years from 1952. From 1973-1992 3,502 Qingke landrace accessions were collected at sites 580 - 4,750 m above sea level and conserved in the Chinese National Gene Bank (Ma, 2000). The area of Qingke cultivation was first divided into three regions, including a winter barley region, a spring barley region and a mixed region (Ma, 2000), and then sub-divided into four ecological zones including a warm humid and semi-humid zone, a warm semi-dry zone, a cold semi-humid zone, and a cold semi-dry zone (Du, 2007). Draft genomes of Qingke landraces “Lhasa Goumang” and cultivar “Zangqing 320” revealed a unique genome structure and gene expression pattern (Zeng et al., 2015; Dai et al., 2018). Genes related to stress resistance, including hormone signal transduction, plant pathogen interaction and secondary metabolic synthesis in Qingke were different from inland barley, suggesting a unique genetic basis for adaptation to harsh plateau environments (Zeng et al., 2015). A recent metabolomic analysis proved that the accumulation of flavonoids in Qingke landraces was significantly higher than in inland barley accessions, suggesting important roles of flavonoids under strong sunlight and ultraviolet radiation (Zeng et al., 2019).

Highland Qingke landraces adapted to the extreme environments of Tibet evolved over long period of domestication, but little is known about the population structure, genomic selection traces and most frequent alleles. A preliminary genome-wide association study (GWAS) using a small number of Qingke landraces and cultivars revealed genetic differences among Qingke cultivars and landraces in three genomic regions affecting heading date (Li et al., 2020). As the population size in that study was too small to gain a broad measure of population structure and genomic differentiation among subpopulations, the present study was expanded to 1,494 accessions (42.7% of the total 3,502) including all sub-types. The aims of the research were to (i) determine the population structure of Qingke landraces; (ii) identify the genetic differentiation among sub-populations; and (iii) determine alleles contributing adaptation of Qingke to highland environments. This study provides information on genome evolution, core germplasm construction and breeding of Qingke in Tibet.

## Results

### SNP analysis by tGBS

We detected 4,686,811,287 reads aligned to the reference genome sequence of barley cv. Morex (*Hordeum vulgare* Hv IBSC PGSB v2) (Ott, et al, 2017). The average and unique mapping rates were 83.1% and 54.5%, respectively. A total 46,719 high-quality SNPs were obtained after filtering according to criteria: MAF ≥1%, marker missing rate ≤30%, and individual missing rate ≤24.5%. We chose 1,366 barley accessions (**Supplemental Table 1**) for analysis. The SNP number and density for each chromosome were tabulated (**Supplemental Table 2**; **Supplemental Figure 1**). Similar segments were present in other chromosomes, but not at very high density (**Supplemental Figure 1**). Genes containing SNPs were annotated based on their positions in the reference genome (**Supplemental Table 3**; **Supplemental Figure 2**).

### Population structure

**Figure 1:**
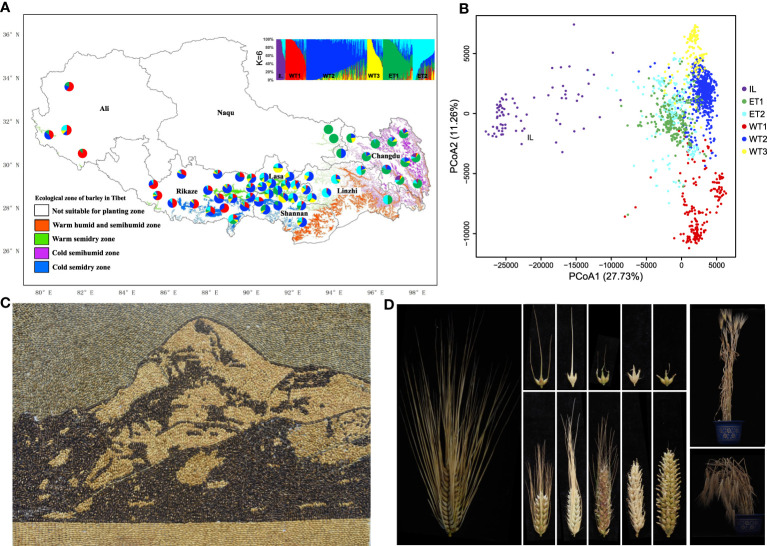
Genetic structure and diversity of Qingke. **(A)** Population structure of Qingke landraces and geographical distribution of six ecotypes. **(B)** Principal coordinates analysis (PCoA) of Qingke landrace accessions. **(C)** Mount Kailash (Gang Rinpoche). It is the most famous mountain in Asian history, located in Ali, Tibet, China. Gang Rinpoche is recognized as a sacred mountain in the world, and it is also recognized as the center of the world by Hinduism, Tibetan Buddhism, Tibetan native religion Bon and ancient Jainism. **(D)** Genetic diversity of panicle traits and plant types of Qingke resources.

Admixture and principal coordinates analysis (PCoA) showed that the inland (IL) and Tibetan (T) groups were significantly separated when *K* = 2. Qingke accessions were further divided into Western Tibetan (WT) and Eastern Tibetan (ET) types when *K* = 3. WT was divided into WT1 (high altitude) and WT2 (central Tibet), whereas ET groups remained basically unchanged when *K* = 4. At *K* = 5, WT2 was further divided into WT2 and WT3, whereas IL, WT1 and ET remained unchanged. *K* = 6 was most informative; all accessions were divided into six groups, ET1, ET2, WT1, WT2, WT3 and IL (**Figure 1**; **Supplemental Figure 3**), closely corresponding to the ecological and cultivation regions in Tibet (Ma, 2000; Du, 2007). The number of accessions in each group varied from 20 to 525, *viz*. IL 20 accessions, WT1 179, WT2 525, WT3 137, ET1 257, and ET2 190 (**Figure 1**). Both PCoA and phylogenetic analysis confirmed the clustering results (**Figure 1**). Qingke accessions comprised two distinct eastern and western clusters reflecting very different climatic conditions on each side of the Mila Mountain range (5,018 m altitude). Eastern Tibet is humid or semi-humid and mainly affected by the Indian Ocean, whereas western Tibet has a typical arid inland climate. Among 1,308 Qingke accessions, 20 IL types were found in three ecological regions including Shannan (one accession), Rikaze (6) and Changdu (13). The other accessions were distributed across four ecological regions (**Supplemental Tables 4.1 and 4.2**). WT1 was predominantly distributed in cold, arid areas in western Tibet; this region had the lowest temperatures, strongest solar radiation and highest altitude (average 4,043.45 m). The frequencies of WT1 accessions varied from 3.0% in Changdu to 62.1% in Ali, tending to increase with rising altitudes above 3,800 m. WT2 and WT3 accessions originated from average altitudes of 3,845 m and 3,661 m, respectively, and were mainly distributed in the warm and semi-arid areas in central Tibet, with frequencies of 70.0% and 66.7% at Lhasa and Shannan, respectively, located in the Yarlung Zangbo River Basin and representing the core cropping region of Tibet. ET1 was mainly distributed in the cold, humid area of eastern Tibet (average altitude 3,598 m) and accounted for 63.7% of accessions from Changdu. ET2 was mainly distributed in the warm, humid to semi humid area in eastern Tibet, accounting for 49.6% accessions from Linzhi. This area has more precipitation and comparatively less solar radiation. In order to investigate the difference in phenotype among the five major groups of Qingke (the IL type was not included due to the small number of samples), we conducted phenotyping at Lhasa and Linzhi (2017-2018) under spring and autumn sown conditions. The five groups had significant differences in grain color, plant type, lodging resistance, and heading date. Most WT1 accessions had blue grain, brittle stalks, frequent lodging, and early heading, whereas ET2 accessions were mostly white grained, late maturing and low lodging (**Figures 1C, D**; **Supplemental Figure 4**; **Supplemental Tables 5.1, 5.2**).

### Genomic diversity

**Figure 2:**
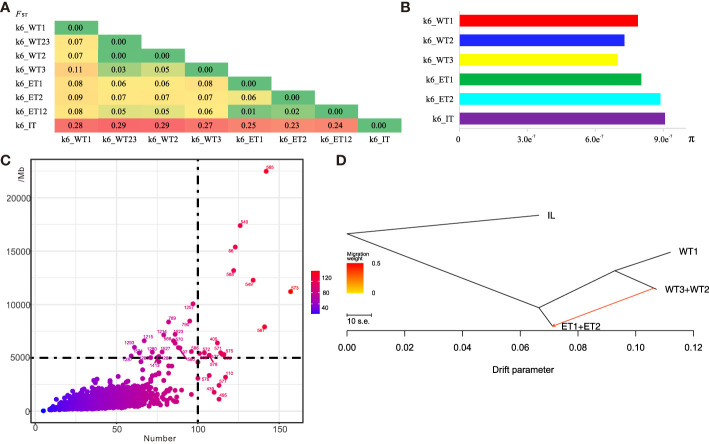
Genome diversity in different types. **(A)** Genetic differentiation (*F*_ST_) of accessions in six Qingke ecotypes. **(B)** Nucleotide diversity(π) of accessions in six Qingke ecotypes. **(C)** IBD (identity by descent) analysis among all the Tibetan samples. **(D)** Genetics flow analysis among accessions. ET (ET1+ET2) appeared first, followed by the WT23 (WT2+WT3) and WT1 ecotypes. Genomic segment exchange occurred between ET and WT23.

Multiple comparisons among the six groups showed that the average *F*_ST_ except for WT1 was close to 0.05 indicating a relatively close relationship between groups (**Figure 2**). When WT1 was compared to other groups *F*_ST_ increased to 0.07-0.11, indicating medium differentiation among the groups (**Figure 2**). When the five Qingke groups were compared with inland barley the differentiation index was much higher with *F*_ST_ ranging from 0.23 to 0.29. ET2 was closest to IL, and WT2 was most the distant (**Figure 2**). There was a low level of genetic diversity with the average π value 8.2 × 10^-7^ (**Figure 2**), indicating a significant evolutionary bottleneck in Qingke. The π value varied among groups, with ET2 having the highest value and WT3 the lowest (**Figure 2**).

Although there was a low level of genetic differentiation among the five groups, genomic regions with higher *F*_ST_ peaks were identified between certain groups. As the *F*_ST_ between ET1 and ET2 was quite small they were integrated into a single ET group. WT2 and WT3 were similarly combined into WT23. Thus, the Qingke accessions were clustered into four groups for genome diversity analysis, i.e., ET, WT1, WT23 and IL, where ET represented the eastern subpopulation, WT23 represented a mixed western subpopulation, WT1 represented a high-altitude western population, and IL represented the inland barley population.

Interestingly, among the IL types, we found 20 Tibetan barleys samples. To analyze the relationship between these materials and the rest of the Qingke accessions, we performed the IBD among all Tibetan barley samples, among which the 20 IL accessions contributed by high level genome segments to the Qingke (**Figure 2**). Among them, the materials such as Suofu (565), Duomangqignke (540), Ziqinke (86), 588,549, Pidamai (573), Bendidongdamai (567) contributed the most, and were presumed to be one of the important candidate donors of the diversity of modern Qingke landrace.

In order to determine whether gene flow occurred among the three Qingke groups, we used TreeMix software and the BITER package to draw evolutionary trees and identify gene flow events. When IL was used as the outgroup (root) the evolutionary tree showed that ET (ET1+ET2) was formed first, and then differentiated into WT23 (WT2+WT3) and WT1 (**Figure 2**). By evaluation of the number of migration events it was evident that when the number of migrations was 1, the f value (model fitness) reached a maximum, indicating a single migration event (**Figure 2**) that occurred between the ET and WT23 groups (**Supplemental Figure 5**). This single germplasm exchange between eastern and mid-western groups over a long period of domestication of Qingke accounted for their high genetic similarity. However, the WT1 group experienced no genetic exchange with other groups and became a relatively independent group adapted to the extreme, high-altitude environment.

**Figure 3:**
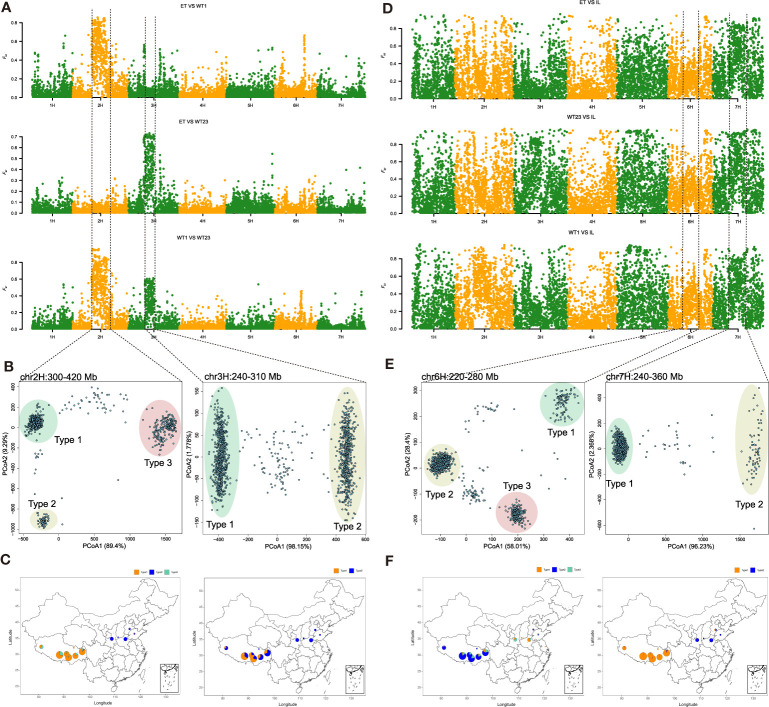
Genetic differentiation among. **(A)** Genetic differentiation among ET, WT23 and WT1 calculated in 100 kb genomic intervals. **(B)** Principal coordinates analysis (PCoA) on the pericentric regions of chromosomes 2H and 3H. **(C)** Geographical distribution of the major pericentric haplotypes of chromosomes 2H and 3H. **(D)** *F_ST_* analysis Qingke compared with inland barley. Extensive differences between the IL type and the other five types at genome wide level. **(E)** Principal coordinates analysis (PCoA) on the pericentric regions of chromosomes 6H and 7H. **(F)** Geographical distribution of the major pericentric haplotypes of chromosomes 6H and 7H.

Genetic differentiation among ET, WT23 and WT1 was calculated in genomic windows of 100 kb. Three highest *F*_ST_ peaks on chromosomes 2H, 3H and 6H were between ET, WT23 and WT1 (**Figure 3**). The highest peak of 6H (399-411 Mb) in the ET vs WT1 comparison overlapped with a previously reported biotic stress resistance related QTL (6H:406-410 Mb) (Novakazi, 2020), which suggested that resistance for biotic stress might play an important role in the ecological diversification of Qingke. The highest peak in the WT1 vs ET comparison was in the pericentric region of 2H (258-491 Mb) (**Figure 3**); between WT23 and ET, there was a high peak at the pericentric region of 3H (208-305 Mb) (**Figure 3**). There were two high peaks in the WT1 vs WT23 comparison (**Figure 3**). It can be speculated that groups ET, WT1 and WT23 have significant differentiation on the pericentric regions of chromosome 3H, and that WT1 has an additional differentiated region on chromosome 2H. Principal coordinates analysis (PCoA) based on genetic distance on the pericentric regions of chromosomes 2H and 3H (**Figure 3**) suggested the presence of three and two major haplotypes, respectively. Meanwhile, a few intermediate individuals with potential recombination between haplotypes were observed. The pericentric haplotypes were highly differentiated between inland barley and Qingke (**Figure 3**). Most inland barley accessions have 2H haplotype 2 (79.5%), while most Qingke accessions belong to either 2H haplotype 1 (79.3%) or to haplotype 3 (16.8%) (**Figure 3**). Similarly, 3H haplotype 1 only presented in Qingke accessions (54.5%) (**Figure 3**). Further analyses showed that the pericentric haplotypes of chromosome 6H and 7H (**Figure 3**) were also highly differentiated between inland barley and Qingke (**Figures 3E, F**). Generally, the highly diversified haplotype across the large pericentromeric regions of 2H, 3H, 6H, 7H might represent the complex origins of these chromosome backbones, similar as the centromere ancestral haploblocks which were recently reported in domesticated wheat (Wang et al., 2022).

Population structure and geographic distribution showed that accessions collected from extremely high altitudes were assigned to WT1. The difference between ET and WT23 was mainly caused by geographic separation of east and west. The differentiated region on chromosome 2H could be related to adaptation to extremely high-altitude conditions in western Tibet characterized by drought and strong ultraviolet radiation, whereas that on 3H was more likely related to the higher precipitation, lower sunshine intensity, and pest and disease resistance in eastern Tibet (**Supplemental Table 6**). According to gene annotation which were downloaded from https://plants.ensembl.org/index.html, genes in the chromosome 2H region were related to photosystem II (**Supplemental Figure 6**; **Supplemental Table 7**). It is generally believed that barley pigments are important factors in adaptation to strong ultraviolet radiation in highland areas, and enrichment of genes on the 2H involved in photosynthesis may contribute to local adaption. The differentiated genomic region on chromosome 3H had significantly enriched genes related to DNA binding, serine metabolism pathway and the protein localization transport pathway (**Supplemental Figure 7**; **Supplemental Table 8**). The translation products of these genes included U-box proteins, protein kinases, and zinc finger proteins. Therefore, we infer that stress resistance genes related to interaction with pathogens, plant hormone signal transduction, and secondary metabolism synthesis might be important for the adaptation of Qingke landraces to highland environments.

### Variation in *HvVRN1*

**Figure 4:**
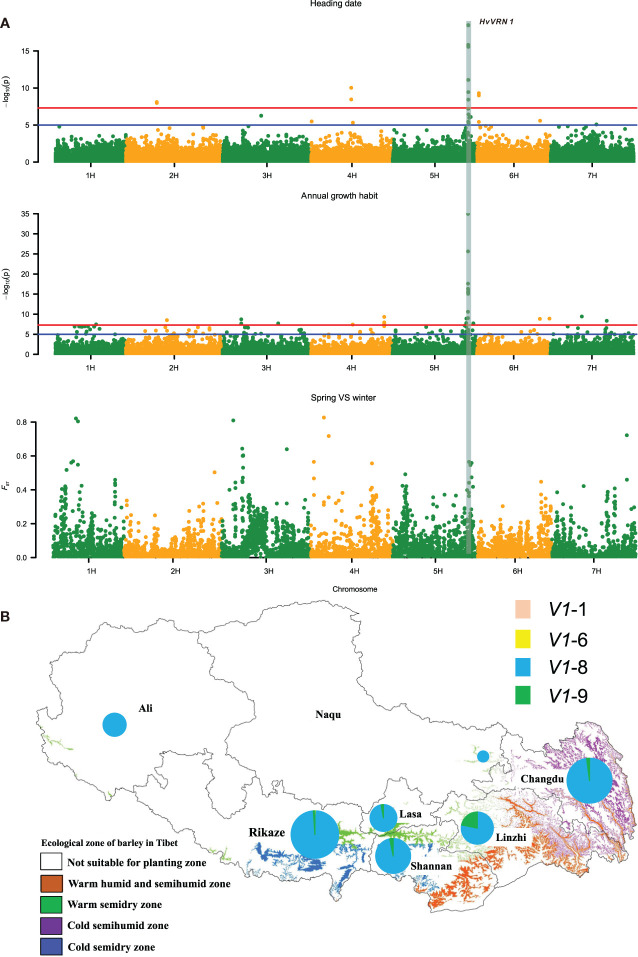
GWAS analysis on annual growth habit and heading date. **(A)** Manhattan plots of GWAS and *F*_ST_ related to heading date and growth habit shows selective sweep sites between winter and spring accessions. The red and blue horizontal lines denote suggestive threshold value *P* < 5×10^-8^ and *P* < 1×10^-5^, respectively. **(B)** Geographical distribution of *HvVRN-1* haplotypes in Qingke landraces.

Growth habit is a key factor in the ecological adaptability of barley. Phenotyping of growth habit following different planting times showed that all inland accessions were winter type, whereas the majority of Qingke landraces (96% of 1,254) were spring types, and only 54 accessions (4%) were winter type (**Supplemental Table 1**). Spring-type Qingke landraces were distributed across all ecological regions whereas those with winter habit were restricted to eastern Tibet (**Supplemental Figure 8**). GWAS identified a locus in genome region 597.5-600 Mb on chromosome 5 significantly associated both with growth habit and heading date (**Figure 4**). *F*_ST_ analysis between winter and spring types confirmed this region, which contained 56 genes, two of them having MADS-box domains, including *HORVU5Hr1G095630* (MADS-box transcription factor 14) and *HORVU5Hr1G095710* (MADS-box transcription factor 34). *HORVU5Hr1G095630* was predicted to be *HvVRN1*. Other genes such as *Ppd-H1*, *HvVRN2* and *HvVRN3* were also found in corresponding *F*_ST_ regions. Several candidate genes on chromosomes 1H, 3H, 4H and 7H were identified, where *F*_ST_ was as high as 0.8; these require further study.

Molecular marker analysis of *HvVRN1* identified 5 haplotypes, including *Hvvrn1* (*v1*), *HvVRN1*-1 (*V1*-1), *HvVRN1*-6 (*V1*-6), *HvVRN1*-8 (*V1*-8) and *HvVRN1*-9 (*V1*-9) (**Figure 4**; **Supplemental Figure 9**). All inland landraces carried haplotype *v1*, whereas most (95.3%) Qingke landraces were haplotype *V1*-8, followed by 52 accessions (4%) with haplotype *V1*-9 and three rare haplotypes *v1* (three accessions), *V1*-1 (1) and *V1*-6 (6) (**Supplemental Table 1**). Haplotype *v1* and *V1*-6 accessions were winter-type whereas haplotype *V1*-1 accessions were spring type, consistent with a previous study (Dondup et al., 2016). Most *V1*-8 accessions were spring-type (2 exceptions) whereas most *V1*-9 accessions were winter-type (8 exceptions). Thus the alleles for spring and winter growth habit in Qinkge landraces were predominantly represented by haplotypes *V1*-8 and *V1*-9, respectively.

### Variation in grain color

Among the 1,308 Tibetan barley accessions, 861 (65.8%) had colored grains. The three major colors (blue, purple and white) can be further divided into 9 sub-colors based on the color level (**Supplemental Figure 10**). For different altitude, the frequency of colored barley totally accounted for more than 50% accessions (**Table 1**). The frequency of blue-grained barley increased with altitude and peaked (52.5%) at 4,440 m, whereas the frequencies of purple and white grained ones decreased with increasing altitude, the frequency of purple grained accessions peaked (51.2%) at the lowest altitude (<2,800 m).

**Table 1:** Frequencies of Qingke landrace accessions with different grain colors.

Altitude	No. of accessions	No. of accessions (frequency)			
		Blue	Purple	Blue + Purple	White
>4400	40	21 (0.53)	12 (0.3)	33(0.83)	7 (0.18)
4200-4400	188	87 (0.46)	52 (0.28)	139(0.74)	49 (0.26)
4000-4200	57	29 (0.51)	10 (0.18)	39(0.69)	18 (0.32)
3800-4000	434	188 (0.43)	109 (0.25)	297(0.68)	137 (0.32)
3600-3800	177	58 (0.33)	45 (0.25)	103(0.58)	74 (0.42)
3400-3600	158	40 (0.25)	53 (0.34)	93(0.59)	65 (0.41)
3200-3400	81	28 (0.35)	22 (0.27)	50(0.63)	31 (0.38)
3000-3200	61	17 (0.28)	20 (0.33)	37(0.61)	24 (0.39)
2800-3000	73	20 (0.27)	24 (0.33)	44(0.60)	29 (0.40)
<2800	39	6 (0.15)	20 (0.51)	26(0.66)	13 (0.33)
Total	1308	494	367	861	447

**Figure 5:**
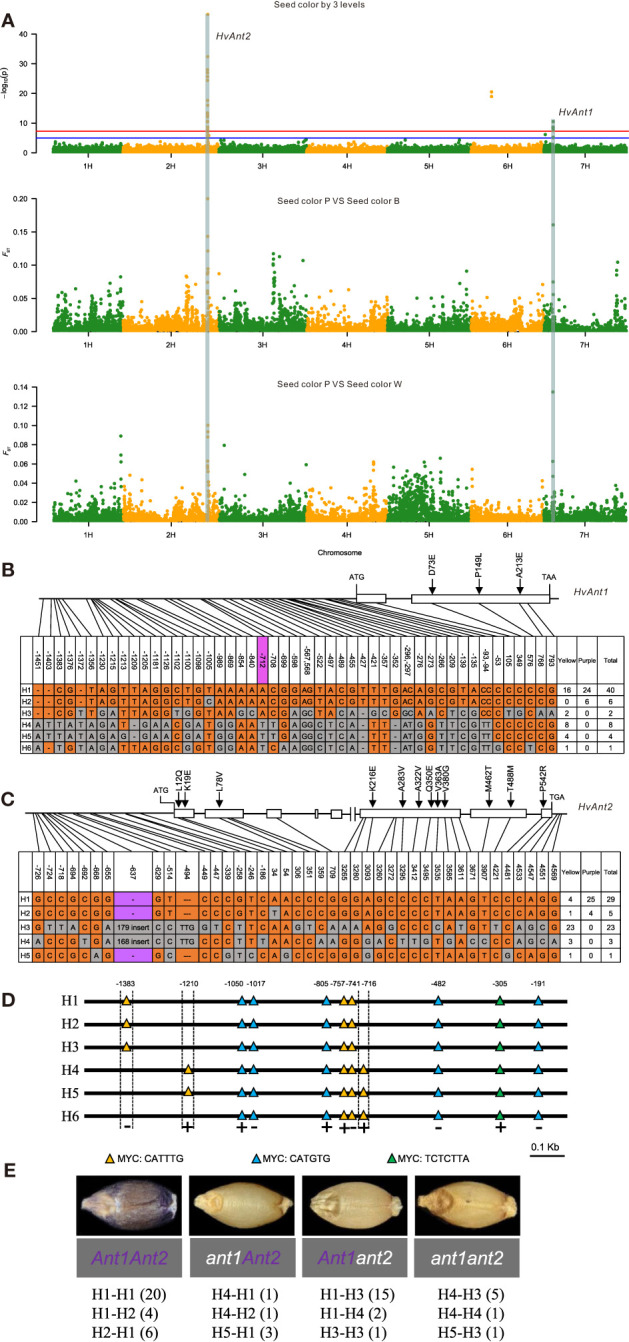
Variation in Qingke grain color. **(A)** GWAS and *F*_ST_ analysis on purple grain color in Qingke landraces. Manhattan plots for GWAS analysis of purple grain. The red and blue horizontal lines denote suggestive threshold value *P*  < 5×10^-8^ and *P* < 1×10^-5^, respectively. The peak regions on chromosome 2HL and 7HS along with *F*_ST_ between purple and blue grain color; The peak regions on chromosome 2HL and 7HS along with *F*_ST_ between purple and white grain color. **(B, C)** Structure of **(B)** 6 haplotypes of *HvAnt1* and **(C)** 5 haplotypes of *HvAnt2* among 30 white grain Qingke accessions and 30 purple grain Qingke accessions. ATG and TGA represent the initiation codon and termination codon, respectively. The haplotype H6 of *HvAnt1* and H5 of *HvAnt2* refer to the Morex reference gene. The numbers in tables indicate accession number of various haplotypes corresponding to different colored Qingke. White boxes indicate exons, solid lines between the white boxes indicates intron. The key causal variant was marked by the color of light violet. **(D)** Promoter cis-elements of *HvAnt1* using online PlantCARE software. That haplotypes H1, H2, and H3 possessed nine MYC sites (CATTTG/CATGTG/TCTCTTA), whereas haplotypes H4 and H5 harbored ten MYC sites. **(E)** Haplotype combinations of complementary genes *HvAnt1* and *HvAnt2* causing purple grain color. Numbers in brackets indicate numbers of re-sequenced Qingke landrace accessions.

Based on the three major colors, GWAS and *F*_ST_ analysis identified two sites on 2HL (674,754,805-676,852,973 bp) and 7HS (72,245,052-73,506,619 bp), which contained the complementary genes *HvAnt1* (7HS) and *HvAnt2* (2HL), identified as MYB and bHLH transcription factors, respectively (**Figure 5**). *HvAnt2* and *HvAnt1* together determine purple grain color in barley (Shoeva et al., 2015; Shoeva et al., 2016; Zhou et al., 2021), To determine how these genes vary in highland barley, the promoters, exons and 3’-UTR regions of both genes were re-sequenced and analyzed in 30 white and 30 purple grain accessions. We identified five haplotypes (H1-H5) for *HvAnt1* and four haplotypes (H1-H4) for *HvAnt2*. Morex had different haplotypes for both genes, classified H6 and H5, respectively (**Figures 5B, C**). Since the polymorphisms in amino acid sequence did not explain the variation in grain color we investigated the promoter regions. The *HvAnt1* and *HvAnt2* promoters were highly conserved in the tested accessions. However, PLANTCARE promoter analysis of *HvAnt1* showed that haplotypes H1, H2, and H3 possessed nine MYC sites (CATTTG), whereas haplotypes H4 and H5 harbored ten (**Figure 5**). The SNP variation at -712 bp upstream of the *HvAnt1* start codon might be the key variant for white grain color. We inferred that haplotypes H4 and H5 of *HvAnt1* were the non-functional haplotypes that led to white grain color. A 179/168 bp insertion in the promoter of *HvAnt2* (H3 and H4) was considered to be the causal variation responsible for the white grain barley. The correlation between haplotype combinations and grain color further verified this deduction. The *HvAnt1* and *HvAnt2* haplotype combinations of the 30 purple grain accessions were *HvAnt1-H1*/*HvAnt2-H1* (twenty accessions), *HvAnt1-H1*/*HvAnt2-H2* (4 accessions), *HvAnt1-H2*/*HvAnt2-H1* (6 accessions). For the white grain accessions, there were 9 haplotype combinations of *HvAnt1* and *HvAnt2* and recessive alleles of any one of the two genes led to white grain color (**Figure 5**). The KASP-SNP marker of *HvAnt1* and the InDel marker for *HvAnt2* distinguished white and the purple grain barley accessions (**Supplemental Figure 11**).

Based on 9 sub-colors, GWAS and *F*_ST_ analysis revealed two sites associated with grain color (**Supplemental Figure 12**), where chromosome arm 4HL (532,216,605-537,053,646 bp) contains gene cluster MbHF35 including *HvMYB4H*, *HvMYC4H* and *HvF35H* responsible for blue grains as reported by Jia et al. (2020), and arm 7HL (504,844,632-505,339,780 bp) contains a locus relating to *Blx2* and *Blx5* (Shim and Suh, 1986), which might also be responsible for the blue grains.

## Discussion

The average altitude of the Tibet plateau is 4,000 m and Qingke is the most widely planted crop. Qingke, distributed between 570 and 4,750 m altitude, covers four ecological environments (**Figure 1**) (Ma, 2000). Much effort has been applied to collect Qingke germplasm, genome sequencing, and gene cloning (Ma, 2000; Zeng et al., 2015; Dai et al., 2018; Jia et al., 2020). Qingke have been cultivated in Tibet for thousands of years, and its genetic structure, genome diversity and origin remain unclear. Our study based on the tGBS identified six types of Tibetan barley landraces, and five of them were Qingke (six-row and naked), i.e. ET1, ET2, WT1, WT2 and WT3, each closely corresponding to ecological and climatic characteristics of the Tibetan plateau. The remaining type belonged to the inland barley type but also contained four accessions named as Qingke. Solar radiation, temperature, and precipitation in different ecological regions of the Tibetan plateau are quite different. From east to west, the solar radiation increases significantly, and the temperature and precipitation decrease significantly (**Supplemental Table 6**). Our results indicated that environmental conditions such as low temperature, high UV and low oxygen are the driving force for the genomic differentiation of subgroups of Qingke landraces. Similar findings on local adaptation in Israeli wild barley were reported by Bian et al. (2020). Different soil ecological environments lead to morphological and genomic differentiation, and wild barley has evolved different phenotypic traits to adapt to different environments (Bian et al., 2020). Mila Mountains (5,018 m altitude, across the Yarlung Zangbo River valley) imposes a separation between east and west in terms of landform, vegetation and climate. The climate to the west is dry and cold, and to the east, warm and humid favoring more plant growth. This is also the major division between eastern (ET) and western (WT) Qingke landraces, with further subdivision according to altitude. In areas with more rainfall in eastern Tibet, Qingke are dominated by late-maturing genotypes with strong stems, whereas in western arid and low-temperature regions, Qingke are dominated by early maturing genotypes with brittle stems. Variation in agronomic traits related to adaptability and yield completely reflect the ecological environment (**Supplemental Table 5.2**).

Large blocks of chromosomal variation in chromosomes 2H and 3H among WT1, WT23 (WT2+WT3) and ET (ET1+ET2) were identified and genes in these regions likely caused the geographic differentiation of eastern and western Qingke. Guo et al. (2020) reported that the high-altitude environment led to the remodeling of Tibetan semi-wild wheat genotypes, and the loss of a region of chromosome 3D including the *Btr* gene could be related to adaptation to the plateau environment. Our research shows that the variation in chromosomes 2H and 3H is associated with the differentiation of different groups. We speculate that changes in the photosystem II response gene family on chromosome 2H, and the protein kinase family and *Btr* locus on 3H were important factors in adaptation to highland environments. The specific genes or gene clusters that promoted adaptation of Qingke to the plateau need to be further studied.

Anthocyanins contribute to plant defense against stress agents (Shoeva et al., 2015). The grain color of barley is an important crop trait under agricultural selection (Long et al., 2019). The abundance of accessions with colored grains is a remarkable characteristic of Qingke. Most of the Qingke landrace preserved in the national gene bank are colored, and 68% of wild barley accessions from Tibet are colored and all wild barley from above 4,000 m has colored grain. In this study the frequency of Qingke landraces with blue or purple grain color was 66% and tended to increase with the rising altitude. Most Qingke landraces from the western high-altitude areas had blue grain color, especially those in WT1. The higher proportion of accessions with blue grain color at altitudes above 3,800 m suggests that the blue color is strongly related to UV resistance (**Table 1**). Similarly, the frequency of Qingke landraces with purple grain color were mostly distributed in altitudes from 2,800 to 3,800 m. It seems that genotypes with purple and blue color may be specifically adapted to different harsh environments.

It is well known that annual growth habit and flowering time are key factors related to survival and environmental adaptation. Flowering to coincide with seasonal conditions is critical in maximizing reproductive success (Amasino, 2010). Relative to the low altitude inland region the climate of Tibet is characterized by lower temperature than that at the same latitude, and there is a wider variation in temperature between day and night. Our study found that *HvVRN-1* was bottlenecked in the evolution of Qingke landraces. Haplotypes *V1-8* and *V1-9* were the two most frequent types in Tibet. Hemming et al. (2009) considered *V1-8* to be one of the main barley genotypic groups outside of Europe. Chinese barley germplasm carrying haplotype *V1-8* were mainly distributed in the Qinghai-Tibet Plateau and only a small number were found in germplasm from northern China. *V1-9* was mainly restricted to winter barley zones in the Sichuan Basin and Southwestern Plateau (Dondup et al., 2016). These two haplotypes are the most frequent types of Qingke landraces in spring and winter growth habit, respectively. Genome-wide association analysis further showed that *HvVRN-1* is the main locus determining annual growth habit and heading date of Qingke, indicating that temperature-related genes, and even genes on the vernalization pathway play an important role in adaptation of Qingke to the Tibetan plateau.

Owing to the long-term planting and evolution of Qingke in complex ecological environment of Tibet plateau, almost all germplasms are six-rowed, naked, spring habit, and variable in grain color. According to PCoA and structure analysis, Qingke and inland barley were clearly distinguished, with extensive differences at the genome-wide level (**Figure 3**). Ten haplotypes of pericentric regions of 2H, 3H, 6H and 7H were identified which not only clearly separated inland barley and Qingke into different sub-populations, but also revealed multiple origins of Tibetan Qingke. Based on genomic sequence and archaeological research, Zeng et al. (2018) and Chen et al. (2015) speculated that northeastern Tibet may be one route of barley introduction into Tibet. We found that 13 of 20 IL accessions came from the Changdu region and most of them were hulled including one two-row accession. Among them, Suofu and Suowa are old landraces grown for a long time. Ma (2000) also reported that most Tibetan hulled barleys were collected in the Changdu region. Therefore, we speculate that that inland barley was an important progenitor of Qingke, and this is supported by the high frequency of haplotype *V1-8* in Qinghai-Tibet Plateau germplasm (Dondup et al., 2016). In addition, there was extensive genetic variation among the 20 IL varieties (**Supplemental Figure 13**), and among them, Sofu might be the oldest progenitor of these inland barleys. The early introduced ancestors presumably had high genetic diversity, and strong natural selection in Tibet and further evolved to reform its own unique and favorable variation type and spread in Tibet. The results of gene flow analysis confirmed that, after IL the ET type appeared first, followed by WT23 and WT1, and there was indeed genetic exchange between ET and WT23 (**Figure 2**). This gene flow assisted exchange between Qingke landraces from the east and west through Buddhist pilgrimage activities (Tibetans have the cultural heritage of worshipping with newly harvested Qingke of the best quality). This result shows that the WT1, WT23 and ET types might have originated through the same transmission pathway, but the exact source is unclear.

## Conclusion

In adapting to the extreme highland environments of the Qinghai-Tibet Plateau, highland barley was bottlenecked and thus has low nucleotide diversity despite long period of domestication and natural selection. However, it is rich in morphological variation, grain color and awns. Two predominant adaptation variations were identified for low temperature tolerance and grain color, where haplotypes *V1-8* and *V1-9* were most frequent among spring and winter Qingke, respectively, and blue grain color tended to predominate at the highest altitudes. The 1,308 highland and 58 inland barley accessions mainly clustered into six sub-populations, including five Qingke types and one inland type, which was very correspondent with its unique environment, respectively. Two significant divergent regions were identified in chromosomes 2H and 3H between Qingke WT1, WT23 and ET. Genetic variation in these regions might have played key roles in forming the three major types of Qingke. Genome-wide variations were observed between the Qingke groups and IL barley. Among IL barley, 20 were newly identified in Tibet highland barley, and several were named as Qingke, indicating that inland barley was an important origin of Tibetan Qingke, but how the other three types of Qingke developed in Tibet requires more studies. These results clearly divided the highland barleys into four major groups and revealed their unique diversity and two predominant highland adaptation variations providing important clues for the origin, genome evolution, highland adaptation and genetic improvement of highland barley.

## Materials and methods

### Materials

A total of 1,308 Tibetan barley landraces (Qingke) along with an additional 58 inland barley landraces were selected for analysis based on geographical distribution, passport information, avoidance of duplication and in proportions commensurate with the Chinese National Gene Bank. The inland barley landraces were collected from Hebei, Shanxi, Shandong, Henan, Jiangsu provinces during 1975-1985. Qingke specifically refers to cultivated six-row, naked barley from Tibet, whereas inland barley includes mostly two-row, hulled accessions.

### Plant selection for genotyping

The materials from the National Gene Bank were planted at Lhasa (91°04′E, 29°64′N, altitude 3,660 m) in April 2017, and a single plant from each accession was selected for genotyping.

### Growth habit

All accessions were planted at two sowing dates (April 5 2018 and April 25 2018) in order to phenotype growth habit. Those heading from both sowing dates were classified as spring type and those failing to head within 100 days from both sowing dates were considered winter type.

### Grain color

Sun-dried seeds of each accession were scored for color, namely white, blue or purple.

### SNP acquisition and annotation

Clean reads were mapped to the Morex reference genome using Bowtie2 (version 2.3.2), which was downloaded from Ensemble Plant. Reads used for further SNP calling were selected by having a unique mapping position in the Morex genome and a mapping score of more than 30. The mapping results were converted to BAM format by SAMtools (version 0.1.18) (Li et al., 2009).

SNP calling was performed using the HaplotypeCaller of GATK 3.8 (https://software.broadinstitute.org/gatk/). SNPs were obtained after filtering using VCFtools according to criteria: MAF ≥1%, marker missing rate ≤30%, and individual missing rate ≤24.5%. The datasets were available in https://www.ncbi.nlm.nih.gov/sra/PRJNA606408. The snpEff package (Cingolani et al., 2012) was used for SNP annotation in the Morex genome sequence.

We statistically analyzed the distribution of all SNPs in exon regions, splicing sites (within 2 bp of a splicing junction), 5’- and 3’-UTRs, intronic regions, upstream and downstream regions (within a 5kb region upstream or downstream from the transcription start site), and intergenic regions. A heat map of SNP distribution on chromosomes was produced using R-package CMplot.

### Population genetic analysis

We performed principal component analysis of the 46,719 SNP matrix for the 1,366 Qingke accessions using the smartpca program in gcta64 software (Price et al., 2006), and the first three principal components were plotted in two dimensions. We also investigated the population structure using ADMIXTURE (v1.3.0) (Alexander and Lange, 2011), specifying *K* values ranging from 2 to 6. The most suitable number of ancestral populations was determined by the *K* value with the lowest cross-validation error (CV).

The diversity indicators of *F*_ST_ and π across the Morex genome were calculated by using VCFtools (version 0.1.12) (Danecek et al., 2011) in 1 Mb windows and 500 kb steps.

### Population genomic segmentation of pericentric haplotypes

The segmentation of pericentric haplotype for chromosome 2H and 3H, 6H, 7H were defined based on *F*_ST_ of ET vs WT1 comparison and ET vs WT23 comparison, respectively. Binary segmentation algorithm implemented in R package changepoint v2.2.2 (Chen and Gupta, 2012) with BIC penalty on the mean change in *F*_ST_ were used with manually adjustment.

SNPs within the regions were used to calculate IBS distance between all pairs of accessions using Plink v1.9 (Purcell et al., 2007). The principal coordinates analysis (PCoA) was performed using the R function “cmdscale” based on the IBS distance matrix. Clusters were defined from PCoA1 and PCoA2 and the genotype of pericentric haplotypes for each individual was determined accordingly.

### Gene flow

Population admixture graphs for the *K* = 3 subpopulation with 20 IL accessions as the outgroup population were inferred using TreeMix (Pickrell and Pritchard, 2012). TreeMix was run with m = 1 through 8, and k = 1, 200, 400, 600, 800 and 1,000. Results computed from different values of k were used as replicates to fit linear models to identify the optimal number of migration episodes using the BITE package on R.

### Genome-wide association analysis

We carried out a GWAS on data for three phenotypic traits and the genotypic data of 46,719 common SNPs (MAF >0.01) with a mixed linear model (MLM) program by EMMAX (Zhou and Stephens, 2012). The false discovery rate (FDR) was calculated for significant associations using the Benjamini and Hochberg (1995) correction method, with 1.0 × 10^-5^ as the threshold. The Manhattan and quantile-quantile plots were generated by the qqman (Turner, 2014) package in R. The candidate genes in these significantly associated loci were identified by BLASTP.

### Identity-by-descent (IBD)

The genomic segments are identical because they were inherited from a common ancestor without recombination. After removing the samples with high individual missing rates (>24.5%), IBD between accessions was calculated using PLINK v1.9 (Purcell et al., 2007). The total segment lengths of one accession were calculated for comparison. We found extensive genomic exchange among the 20 IL accessions.

### Haplotype analysis of *HvVRN1*

We used the molecular markers reported by Dondup et al. (2016) to identify *HvVRN1* alleles in all samples. Primer information is provided in **Supplemental Table 9**.

### Haplotype analysis of the *HvAnt1* and *HvAnt2* loci

Primer3 online software (https://bioinfo.ut.ee/primer3/) was used to design specific primers for gene promoter regions, coding regions and 3’-UTR regions based on the Morex V3 reference genome. The primers are listed in Appendix **Table 1**. PCR amplification was performed using 2× Taq Plus Master Mix II (Vazyme Biotech, Nanjing), and the PCR products were sent to Sangon Bioengineering (Shanghai) Company for Sanger sequencing. Sequence splicing was performed using Seqman software in DNAstar; DNA sequence alignment was performed using DNAMAN software. Haplotype analysis was performed with DnaSP v5 (Librado and Rozas, 2009). Promoters were analyzed using online PlantCARE software.

### Development of diagnostic markers for *HvAnt1* and *HvAnt2* genes in purple barley

KASP markers were designed and developed from single SNP variant sites in *HvAnt1* and InDel variation in *HvAnt2*. The primer information is provided in in **Supplemental Table 10**.

The PCR system for KASP genotyping consisted 2× Master Mix 96/384, High Rox, (LGC Genomics, Middlesex, UK). PCR were performed in an Applied Biosystems^®^ Veriti^®^ 384-Well Thermal Cycler. The fluorescent signals of PCR products were detected by BMG Omega F (LGC Genomics, UK), and Kluster Caller software was used to perform the statistics.

## Data availability statement

The original contributions presented in the study are included in the article/**Supplementary Material**. Further inquiries can be directed to the corresponding authors.

## Author contributions

DD, JZ, GG, ZQ, and YY designed the project; DD, LN, DX, LG, TD, NK, LD, ZG (11th author), ZS, ZG (13th author), WM, PZ, XT, GJ, WL, ZL, YW, and CW performed the experiments; DD, YY, DX, LN, ZW, YT, and XZ performed the data analysis; DD, YY, DX, WG, GG, and ZQ wrote the manuscript. All authors contributed to the article and approved the submitted version.

## References

[B1] Åberg E. (1938). Hordeum agriocrithon nova sp., a wild six-rowed barley. Ann. Agric. Coll. Sweden 6, 159–121.

[B2] Alexander D. H. Lange K. (2011). Enhancements to the ADMIXTURE algorithm for individual ancestry estimation. BMC Bioinf. 12, 1–6. doi: 10.1186/1471-2105-12-246 PMC3146885 21682921

[B3] Amasino R. (2010). Seasonal and developmental timing of flowering. Plant J. 61, 1001–1013. doi: 10.1111/j.1365-313X.2010.04148.x 20409274

[B4] Benjamini Y. Hochberg Y. (1995). Controlling the false discovery rate: a practical and powerful approach to multiple testing. J. R. Stat. Soc: Ser. B (Methodological) 57, 289–300. doi: 10.1111/j.2517-6161.1995.tb02031.x

[B5] Bian J. Cui L. Wang X. Yang G. Huo F. Ling H.. (2020). Genomic and phenotypic divergence in wild barley driven by microgeographic adaptation. Adv. Sci. 7, 2000709. doi: 10.1002/advs.202000709 PMC7740101 33344112

[B6] Chen F. Dong G. Zhang D. Liu X. Jia X. An C.. (2015). Agriculture facilitated permanent human occupation of the Tibetan plateau after 3600 B.P. Science 347, 248–250. doi: 10.1126/science.1259172 25593179

[B7] Chen J. Gupta A. K. (2012). Parametric statistical change point analysis (Boston: Birkhauser).

[B8] Chen F. Welker F. Shen C. Bailey S. E. Bergmann I. Davis S.. (2019). A late middle pleistocene denisovan mandible from the Tibetan plateau. Nature 569, 409–412. doi: 10.1038/s41586-019-1139-x 31043746

[B9] Cingolani P. Platts A. Wang L. Coon M. Nguyen T. Wang L.. (2012). A program for annotating and predicting the effects of single nucleotide polymorphisms, SnpEff: SNPs in the genome of drosophila melanogaster strain w1118; iso-2; iso-3. Fly (Austin) 6 (2), 80–92. doi: 10.4161/fly.19695 22728672 PMC3679285

[B10] Dai F. Chen Z. Wang X. Li Z. Jin G. Wu D.. (2014). Transcriptome profiling reveals mosaic genomic origins of modern cultivated barley. Proc. Natl. Acad. Sci. U.S.A 111, 13403–13408. doi: 10.1073/pnas.1414335111 25197090 PMC4169977

[B11] Dai F. Nevo E. Wu D. Comadran J. Zhou M. Qiu L.. (2012). Tibet Is one of the centers of domestication of cultivated barley. Proc. Natl. Acad. Sci. U.S.A 109, 16969–16973. doi: 10.1073/pnas.1215265109 23033493 PMC3479512

[B12] Dai F. Wang X. Zhang X. Chen Z. Nevo E. Jin G.. (2018). Assembly and analysis of a qingke reference genome demonstrate its close genetic relation to modern cultivated barley. Plant Biotechnol. J. 16, 760–770. doi: 10.1111/pbi.12826 28871634 PMC5814578

[B13] Danecek P. Auton A. Abecasis G. Albers C. A. Banks E. DePristo M. A.. (2011). The variant call format and VCFtools. Bioinformatics 15, 2156–2158. doi: 10.1093/bioinformatics/btr330 PMC3137218 21653522

[B14] Dondup D. Dong G. Xu D. Zhang L. Zha S. Yuan X.. (2016). Allelic variation and geographic distribution of vernalization genes *HvVRN1* and *HvVRN2* in Chinese barley germplasm. Mol. Breed 36, 11. doi: 10.1007/s11032-016-0434-6

[B15] Du J. (2007). Tibet Agroclimatic resources zone (Beijing: China Meteorological Press).

[B16] Guedes J. D. Lu H. Hein A. Schmidt A. H. (2015). Early evidence for the use of wheat and barley as staple crops on the margins of the Tibetan plateau. Proc. Natl. Acad. Sci. U.S.A 112, 5625–5630. doi: 10.1073/pnas.1423708112 25902511 PMC4426421

[B17] Guo W. Xin M. Wang Z. Yao Y. Hu Z. Song W.. (2020). Origin and adaptation to high altitude of Tibetan semi-wild wheat. Nat. Commun. 11, 5085. doi: 10.1038/s41467-020-18738-5 33033250 PMC7545183

[B18] Hemming M. N. Fieg S. Peacock W. J. Dennis E. S. Trevaskis B. (2009). Regions associated with repression of the barley (Hordeum vulgare) VERNALIZATION1 gene are not required for cold induction. Mol. Genet. Genom 282, 107–117. doi: 10.1007/s00438-009-0449-3 19404679

[B19] Huerta-Sánchez E. Jin X. Asan Bianba Z. Peter B. M. Vinckenbosch N.. (2014). Altitude adaptation in tibetans caused by introgression of denisovan-like DNA. Nature 512, 194–197. doi: 10.1038/nature13408 25043035 PMC4134395

[B20] Jia Y. Selva C. Zhang Y. Li B. McFawn L. A. Broughton S.. (2020). Uncovering the evolutionary origin of blue anthocyanins in cereal grains. Plant J. 101, 1057–1074. doi: 10.1111/tpj.14557 31571294

[B21] Li H. Handsaker B. Wysoker A. Fennell T. Ruan J. Homer N.. (2009). The sequence Alignment/Map format and SAM tools. Bioinformatics 25, 2078–2079. doi: 10.1093/bioinformatics/btp352 19505943 PMC2723002

[B22] Li Z. Lhundrup N. Guo G. Dol K. Chen P. Gao L.. (2020). Characterization of genetic diversity and genome-wide association mapping of three agronomic traits in qingke barley (Hordeum vulgare l.) in the qinghai-Tibet plateau. Front. Genet. 11. doi: 10.3389/fgene.2020.00638 PMC7351530 32719715

[B23] Li M. Tian S. Jin L. Zhou G. Li Y. Zhang Y.. (2013). Genomic analyses identify distinct patterns of selection in domesticated pigs and Tibetan wild boars. Nat. Genet. 45, 1431–1439. doi: 10.1038/ng.2811 24162736

[B24] Librado P. Rozas J. (2009). DnaSP v5: a software for comprehensive analysis of DNA polymorphism data. Bioinformatics 25, 1451–1452. doi: 10.1093/bioinformatics/btp187 19346325

[B25] Long Z. Jia Y. Tan C. Zhang X. Angessa T. Broughton S.. (2019). Genetic mapping and evolutionary analyses of the black grain trait in barley. Front. Plant Sci. 9. doi: 10.3389/fpls.2018.01921 PMC6331406 30671073

[B26] Ma D. ,. Q. (2000). Genetic resource of Tibetan barley in China (Beijing: China Agriculture Press).

[B27] Novakazi F. (2020). Identification of QTL for resistance against two fungal pathogens, pyrenophora teres f. teres and bipolaris sorokiniana, in a barley (*Hordeum vulgare* l.) diversity set. PhD thesis, The Agricultural University of Iceland, Borgarnes, Iceland. doi: 10.5073/dissjki.2020.006

[B28] Ott A. Liu S. Schnable J. C. Yeh C. T. E. Wang K. S. Schnable P. S. (2017). tGBS® genotyping-by-sequencing enables reliable genotyping of heterozygous loci. Nucleic Acids Res. 45 (21), e178. doi: 10.1093/nar/gkx853 29036322 PMC5716196

[B29] Pickrell J. K. Pritchard J. K. (2012). Inference of population splits and mixtures from genome-wide allele frequency data. PloS Genet. 8 (11):e1002967. doi: 10.1371/journal.pgen.1002967 23166502 PMC3499260

[B30] Pourkheirandish M. Hensel G. Kilian B. Senthil N. Chen G. Sameri M.. (2015). Evolution of the grain dispersal system in barley. Cell 162, 527–539. doi: 10.1016/j.cell.2015.07.002 26232223

[B31] Pourkheirandish M. Kanamori H. Wu J. Sakuma S. Blattner F. R. Komatsuda T. (2018). Elucidation of the origin of “agriocrithon” based on domestication genes questions the hypothesis that Tibet is one of the centers of barley domestication. Plant J. 94, 525–534. doi: 10.1111/tpj.13876 29469199

[B32] Price A. L. Patterson N. J. Plenge R. M. Weinblatt M. E. Shadick N. A. Reich D. (2006). Principal components analysis corrects for stratification in genome-wide association studies. Nat. Genet. 38, 904–909. doi: 10.1038/ng1847 16862161

[B33] Purcell S. Neale B. Todd-Brown K. Thomas L. Ferreira M. A. Bender D.. (2007). PLINK: a tool set for whole-genome association and population-based linkage analyses. Am. J. Hum. Genet. 81, 559–575. doi: 10.1086/519795 17701901 PMC1950838

[B34] Qiu Q. Wang L. Wang K. Yang Y. Ma T. Wang Z.. (2015). Yak whole-genome resequencing reveals domestication signatures and prehistoric population expansions. Nat. Commun. 6, 10283. doi: 10.1038/ncomms10283 26691338 PMC4703879

[B35] Qiu Q. Zhang G. Ma T. Qian W. Wang J. Ye Z.. (2012). The yak genome and adaptation to life at high altitude. Nat. Genet. 44, 946–949. doi: 10.1038/ng.2343 22751099

[B36] Shao Q. Q. (1982). Tibet Wild barley (Beijing: China Science Press).

[B37] Shim J. W. Suh S. J. (1986). “Linkage relationship of blue aleurone genes in barley,” in Barley genetics v. proceedings of the fifth international bar- ley genetics symposium. Eds. Yasuda S. Konishi T. (Okayama, Japan: Sanyo Press Co), 213–217.

[B38] Shoeva O. Y. Kukoeva T. V. Borner A. Khlestkina E. K. (2015). Barley *Ant1* is a homolog of maize *C1* and its product is part of the regulatory machinery governing anthocyanin synthesis in the leaf sheath. Plant Breed 134, 400–405. doi: 10.1111/pbr.12277

[B39] Shoeva O. Y. Mock H. P. Kukoeva T. V. Borner A. Khlestkina E. K. (2016). Regulation of the flavonoid biosynthesis pathway genes in purple and black grains of hordeum vulgare. PloS One 11 (10), e0163782. doi: 10.1371/journal.pone.0163782 27706214 PMC5051897

[B40] Simonson T. S. Yang Y. Huff C. D. Yun H. Qin G. Witherspoon D. J.. (2010). Genetic evidence for high altitude adaptation in Tibet. Science 329, 72–75. doi: 10.1126/science.1189406 20466884

[B41] Song S. Y. (2013). Tibet Climate (Beijing: China Meteorological Press).

[B42] Turner S. D. (2014). Qqman: an r package for visualizing gwas results using q-q and manhattan plots. Biorxiv. 005165. doi: 10.1101/005165

[B43] Wang M. Li Y. Peng M. Zhong L. Wang Z. Li Q.. (2015). Genomic analyses reveal potential independent adaptation to high altitude in Tibetan chickens. Mol. Biol. Evol. 32, 1880–1889. doi: 10.1093/molbev/msv071 25788450

[B44] Wang X. Liu S. Zuo H. Zheng W. Zhang S. Pingcuo G.. (2021b). Genomic basis of high-altitude adaptation in Tibetan prunus fruit trees. Curr. Bio 31, 3848–3860. doi: 10.1016/j.cub.2021.06.062 34314676

[B45] Wang Z. Wang W. Xie X. Wang Y. Yang Z. Peng H.. (2022). Dispersed emergence and protracted domestication of polyploid wheat uncovered by mosaic ancestral haploblock inference. Nat. Commun. 13, 3891. doi: 10.1038/s41467-022-31581-0 35794156 PMC9259585

[B46] Xu T. W. (1982). Origin and evolution of cultivated barley in China. Acta Genet. Sin. 9, 440–446.

[B47] Yang Y. T. (1983). Tibet Geography (Beijing: China Science Press).

[B48] Zeng X. Guo Y. Xu Q. Mascher M. Guo G. Li S.. (2018). Origin and evolution of qingke barley in Tibet. Nat. Commun. 9, 5433. doi: 10.1038/s41467-018-07920-5 30575759 PMC6303313

[B49] Zeng X. Long H. Wang Z. Zhao S. Tang Y. Huang Z.. (2015). The draft genome of Tibetan hulless barley reveals adaptive patterns to the high stressful Tibetan plateau. Proc. Natl. Acad. Sci. U.S.A. 112, 1095–1100. doi: 10.1073/pnas.1423628112 25583503 PMC4313863

[B50] Zeng X. Yuan H. Dong X. Peng M. Jing X. Xu Q.. (2019). Genome-wide dissection of co-selected UV-b responsive pathways in the UV-b adaptation of qingke. Mol. Plant 13, 112–127. doi: 10.1016/j.molp.2019.10.009 31669581

[B51] Zhang T. Qiao Q. Novikova P. Y. Wang Q. Yue J. Guan Y.. (2019). Genome of crucihimalaya himalaica, a close relative of arabidopsis, shows ecological adaptation to high altitude. Proc. Natl. Acad. Sci. U.S.A. 116, 7137–7146. doi: 10.1073/pnas.1817580116 30894495 PMC6452661

[B52] Zhou X. Stephens M. (2012). Genome-wide efficient mixed-model analysis for association studies. Nat. Genet. 44 (7), 821–824. doi: 10.1038/ng.2310 22706312 PMC3386377

[B53] Zhou X. Yu J. Spengler R. Shen H. Zhao K. Ge J.. (2020). 5,200-year-old cereal grains from the eastern Altai mountains redate the trans-Eurasian crop exchange. Nat. Plants 6, 78–87. doi: 10.1038/s41477-019-0581-y 32055044

[B54] Zhou C. Zeng Z. Suo J. Li X. Bian H. Wang J.. (2021). Manipulating a single transcription factor, *Ant1*, promotes anthocyanin accumulation in barley grains. J. Agric. Food Chem. 69 (18), 5306–5317. doi: 10.1021/acs.jafc.0c0814 33908247

