# Supplementary material for: Genome diversity and highland-adaptative variation in Tibet barley landrace population of China
Source: Front Plant Sci. 2023 May 10;14:1189642. doi: 10.3389/fpls.2023.1189642 (PMC10206316; doi:10.3389/fpls.2023.1189642)
Supplement: Supplementary file 2 [file Table_2.docx]

**Supplemental Table 2. SNP number and density on each barley chromosome**

| Chromosome | Length | SNP number |
| --- | --- | --- |
| 1H | 558,535,432 | 5,521 |
| 2H | 768,075,024 | 8,001 |
| 3H | 699,711,114 | 7,265 |
| 4H | 647,060,158 | 4,883 |
| 5H | 670,030,160 | 6,226 |
| 6H | 583,380,513 | 6,414 |
| 7H | 657,224,000 | 7,724 |
| *Mt* | *525,599* | *1* |
| *Un* | *249,774,706* | *684* |
| Total | 539,349,410 | 46,719 |

Distribution of all filtered SNPs on 7 chromosomes; the number on 2H is the largest, the number on 1H is the least; chromosome 2H is the longest, and 1H is the shortest. *Mt*, mitochondrial DNA; *Un*, run assembled sequences.

**Supplemental Table 3. Statistics of SNP annotation on the genome**

| SNP type | Count | Percent |
| --- | --- | --- |
| 3_prime_UTR_variants | 2,052 | 1.88% |
| 5_prime_UTR_premature_start_codon_gain_variants | 174 | 0.16% |
| 5_prime_UTR_variants | 1,060 | 0.97% |
| Downstream_gene_variants | 22,223 | 20.37% |
| Intergenic_regions | 43,461 | 39.83% |
| Intron_variants | 15,174 | 13.91% |
| Missense_variants | 1,774 | 1.63% |
| Non_coding_transcript_exon_variants | 415 | 0.38% |
| Splice_acceptor_variants | 5 | 0.01% |
| Splice_donor_variants | 19 | 0.02% |
| Splice_region_variants | 234 | 0.21% |
| Stops_gained | 30 | 0.03% |
| Stops_lost | 1 | 0.00% |
| Synonymous_variantss | 1,934 | 1.77% |
| Upstream_gene_variants | 20,564 | 18.85% |

**Supplemental Table 4.1 Proportions of Qingke landraces collected from different regions within the same group. Majority groups are in bold text**

| Group | No. of samples | Origin | | | | | | | |
| --- | --- | --- | --- | --- | --- | --- | --- | --- | --- |
|  |  | Inland | Ali | Rikaze | Lasa | Shannan | Linzhi | Changdu | Naqu |
| WT1 | 179 | 0.00 | 0.10 | **0.70** | 0.02 | 0.11 | 0.03 | 0.04 | 0.00 |
| WT2 | 525 | 0.00 | 0.01 | **0.49** | 0.12 | **0.27** | 0.06 | 0.06 | 0.00 |
| WT3 | 137 | 0.00 | 0.01 | 0.18 | 0.10 | **0.50** | 0.10 | 0.11 | 0.00 |
| ET1 | 257 | 0.00 | 0.01 | 0.12 | 0.05 | 0.14 | 0.07 | **0.61** | 0.01 |
| ET2 | 190 | 0.00 | 0.01 | 0.16 | 0.08 | **0.27** | **0.35** | 0.13 | 0.00 |
| IL | 78 | **0.74** | 0.00 | 0.08 | 0.00 | 0.01 | 0.00 | 0.17 | 0.00 |
| Total | 1,366 | 58 | 29 | 473 | 107 | 319 | 133 | 245 | 2 |

**Supplemental Table 4.2 Proportions of Qingke landraces collected from the same region distributed in different 6 groups**

| Group | No. of samples | Origin | | | | | | | |
| --- | --- | --- | --- | --- | --- | --- | --- | --- | --- |
|  |  | Inland | Ali | Rikaze | Lasa | Shannan | Linzhi | Changdu | Naqu |
| WT1 | 179 | 0.00 | **0.62** | **0.26** | 0.04 | 0.06 | 0.05 | 0.03 | 0.00 |
| WT2 | 525 | 0.00 | 0.17 | 0.54 | **0.57** | **0.45** | 0.23 | 0.12 | 0.00 |
| WT3 | 137 | 0.00 | 0.03 | 0.05 | 0.13 | **0.22** | 0.11 | 0.06 | 0.00 |
| ET1 | 257 | 0.00 | 0.10 | 0.06 | 0.12 | 0.11 | 0.13 | **0.64** | 1.00 |
| ET2 | 190 | 0.00 | 0.07 | 0.07 | 0.14 | 0.16 | **0.50** | 0.10 | 0.00 |
| IL | 78 | **1.00** | 0.00 | 0.01 | 0.00 | 0.00 | 0.00 | 0.05 | 0.00 |
| Total | 1,366 | 58 | 29 | 473 | 107 | 319 | 133 | 245 | 2 |

**Supplemental Table 5.1 Numbers of Qingke landrace accessions with different grain colors**

| Group | Blue | Purple | White | Total |
| --- | --- | --- | --- | --- |
| WT1 | 97 | 45 | 37 | 179 |
| WT2 | 232 | 116 | 177 | 525 |
| WT3 | 31 | 67 | 39 | 137 |
| ET1 | 87 | 85 | 85 | 257 |
| ET2 | 37 | 54 | 99 | 190 |
| Total | 484 | 367 | 437 | 1,288 |

1,288 naked barley (Qingke) accessions.

**Supplemental Table 5.2 Stem types of Qingke landrace accessions**

| Group | Erect  stem | Necking | Stem  lodging | Root lodging | Mixed  (Stem lodging+  root lodging) | Total |
| --- | --- | --- | --- | --- | --- | --- |
| WT1 | 38 | 20 | 38 | 41 | 42 | 179 |
| WT2 | 294 | 52 | 62 | 69 | 48 | 525 |
| WT3 | 106 | 3 | 12 | 11 | 5 | 137 |
| ET1 | 179 | 24 | 11 | 37 | 6 | 257 |
| ET2 | 157 | 7 | 5 | 12 | 9 | 190 |
| Total | 774 | 106 | 128 | 170 | 110 | 1,288 |

Erect: No lodging on the whole stem.

Necking: Buckling of the peduncle below the ear.

Stem lodging is usually caused by one of the bottom two internodes buckling and results in the upper stem and ear lying horizontally.

Root lodging results in permanent displacement of the cereal stems without any observable stem buckling.

**Supplemental Table 6. Meteorology for five Qingke groups**

| Group | AAT | AAR | AAS | No. of MOS |
| --- | --- | --- | --- | --- |
| WT1 | 8.5 | 219.8 | 1901.4 | 4 |
| WT2 | 9.9 | 354.2 | 1705.7 | 8 |
| WT3 | 12.9 | 441.5 | 1781.0 | 4 |
| ET1 | 10.5 | 477.5 | 1399.0 | 8 |
| ET2 | 14.0 | 627.0 | 1001.6 | 5 |

AAT: Annual average temperature (°C)；

AAR: Annual average rainfall (mm)

AAS: Annual average sunshine (days)

MOS: Meteorological observation stations

**Supplemental Table** **7. Enriched genes related to photosynthesis in the differentiated 2H region**

| Gene name | Start position | End position | Description |
| --- | --- | --- | --- |
| *HORVU2Hr1G048390* | 270,702,335 | 270,702,563 | Photosystem II protein H |
| *HORVU2Hr1G048400* | 270,702,660 | 270,702,788 | Protein PsbN |
| *HORVU2Hr1G057700* | 371,442,220 | 371,443,669 | Oxygen-evolving enhancer protein 1, chloroplastic |
| *HORVU2Hr1G060480* | 403,885,878 | 403,887,371 | Photosystem I subunit psaK, chloroplastic |
| *HORVU2Hr1G060880* | 406,850,365 | 406,851,259 | Oxygen-evolving enhancer protein 2, chloroplastic |
| *HORVU2Hr1G061990* | 419,324,464 | 419,324,877 | Photosystem II D2 protein |
| *HORVU2Hr1G062000* | 419,327,807 | 419,328,019 | Photosystem II protein Z |
| *HORVU2Hr1G062030* | 419,356,666 | 419,358,013 | Photosystem II CP43 protein |
| *HORVU2Hr1G062050* | 419,362,944 | 419,363,149 | Photosystem II protein D1 |
| *HORVU2Hr1G062060* | 419,363,398 | 419,364,111 | Photosystem II protein D1 |

**Supplemental Table 8. Enriched genes related to resistance in the differentiated 3H region**

| Gene name | Start position | End position | Description |
| --- | --- | --- | --- |
| *HORVU3Hr1G037450* | 212,797,245 | 212,798,441 | RING/U-box superfamily protein |
| *HORVU3Hr1G038000* | 217,755,874 | 217,757,764 | Protein kinase superfamily protein |
| *HORVU3Hr1G038320* | 221,036,554 | 221,038,791 | Phosphatidylinositol 4-kinase gamma 1 |
| *HORVU3Hr1G038690* | 224,332,864 | 224,334,426 | Serine/threonine-protein kinase |
| *HORVU3Hr1G039200* | 231,184,853 | 231,186,395 | Pyruvate kinase family protein |
| *HORVU3Hr1G039210* | 231,186,998 | 231,187,722 | RING/U-box superfamily protein |
| *HORVU3Hr1G039220* | 231,377,447 | 231,384,751 | RING/U-box superfamily protein |
| *HORVU3Hr1G039230* | 231,379,567 | 231,384,772 | Pyruvate kinase family protein |
| *HORVU3Hr1G039550* | 233,988,500 | 233,993,909 | DOF zinc finger protein 1 |
| *HORVU3Hr1G039610* | 235,359,531 | 235,360,131 | LRR receptor-like serine/threonine-protein kinase EFR |
| *HORVU3Hr1G040410* | 245,038,947 | 245,039,441 | Protein kinase superfamily protein |
| *HORVU3Hr1G040580* | 246,957,330 | 246,967,525 | Serine/threonine-protein kinase-like protein CCR4 |
| *HORVU3Hr1G041030* | 254,833,576 | 254,843,549 | RING/U-box superfamily protein |
| *HORVU3Hr1G041430* | 256,959,596 | 256,961,836 | Leucine-rich repeat protein kinase family protein |
| *HORVU3Hr1G041810* | 261,010,851 | 261,012,998 | Receptor-like protein kinase 1 |
| *HORVU3Hr1G041820* | 261,022,818 | 261,027,266 | Protein kinase superfamily protein |
| *HORVU3Hr1G042900* | 273,478,386 | 273,479,225 | General transcription factor 2-related zinc finger protein |
| *HORVU3Hr1G043300* | 279,060,631 | 279,068,693 | Zinc induced facilitator-like 2 |
| *HORVU3Hr1G045640* | 299,554,773 | 299,555,615 | Serine/threonine-protein kinase |
| *HORVU3Hr1G046280* | 307,953,499 | 307,971,846 | MORC family CW-type zinc finger protein 3 |
| *HORVU3Hr1G047160* | 319,229,451 | 319,264,381 | Protein kinase family protein / protein phosphatase 2C（PP2C）family protein |

**Supplemental Table 9. Primers used for identifying haplotpes of *HvVRN1***

| Haplotype | Primer ID | Primer sequence (3’-5’) | Product size  (bp) | Tm (℃) |
| --- | --- | --- | --- | --- |
| V1-1 | 5.2F | GTGCTTGCTGCTTGAACAAA | 753 | 55 |
|  | 5.2R | CTAACCGTCGTGGATCGTTT |  |  |
| V1-2 | 6.4F | GCTCCAGCTGATGAAACTCC | 837 | 60 |
|  | 6.4R | TCAACCTGGTCACCATCAAA |  |  |
| V1-3 | 8.9F | CTGTTTCCTTCGGATTCTGC | 700 | 60 |
|  | 8.9R | CACAGGGAGAGGACGAGGTA |  |  |
| V1-4 | 4.1F | AAAAGACCGCCATGAACATC | 479 | 55 |
|  | 4.1R | GTCGACCGCAATCAGTTTTT |  |  |
| V1-5 | 3.9F | GTCGGCCTCATCATCTTCTC | 502 | 58 |
|  | 3.9R | GCGCTTGTGTGTGCATCTAT |  |  |
| V1-6 | 0.5F | CTTGCATGTGTTGTCGGTCT | 537 | 58 |
|  | 0.5R | AAAGCTCCTGCCAACTACGA |  |  |
| V1-8 | 6.6F | TTCTTGCGTAAGGCTGACACAGG | 582 | 60 |
|  | 6.6R | CTCTCCGTCCTCAGCCAC |  |  |
| V1-9 | 2.4F | CGAGAGAGACGCACGATGTA | 677 | 58 |
|  | 2.4R | AAAGGTTTAGCCCCGAAGAA |  |  |
| V1-10 | 5.7F | ACCCCAAGTGGAAGGGTTAG | 612 | 60 |
|  | 5.7R | TCCAGCAACGAGCTTATGTG |  |  |

**Supplemental Table 10. Primers for sequencing *HvAnt1* and *HvAnt2***

| Gene | Forward primer sequence | Reverse primer sequence | Purpose |
| --- | --- | --- | --- |
| *HvAnt1* | CCACGTGGTAATTCTCTGGAG | GAAGGCTTCCATGTGTGCA | PCR amplification of *HvAnt1* |
|  | CTTGCTACGAAAAGTCAACATGT | ACGCACAGTTTCCCTTTGAG | PCR amplification of *HvAnt1* |
|  | GAAGGTGACCAAGTTCATGC  TGACTTTCTTTATTCGGAACT | AGCTGAGTTTCATTTGCCACGTGTG | KASP primers for diagnostic molecular marker |
|  | GAAGGTCGGAGTCAACGGAT  TGACTTTCTTTATTCGGAACA |  |  |
| *HvAnt2* | AAGTCTCTCCTGAATGCCACA | CGAGGTGAAGTTAGAGCAAGC | PCR amplification of *HvAnt2* |
|  | CAGTGCAACAGAGTGGAGGGATT | TGCTTGATGCGACAAAGCGTTAC | PCR amplification of *HvAnt2* |
|  | GACGAACACCCCATCTATCCA | ACGATCTCTTCCAAGCCGTAA | PCR amplification of *HvAnt2* |
|  | CTGGATCATAGGCGGGTCTT | GTCCCACATCACTGCACATC | PCR amplification of *HvAnt2* |
|  | GATTATTCTGTGCCTGTTACCCC | TCCGAGCAACGAGCCAACA | Diagnostic molecular marker |
