# Supplementary material for: Genome diversity and highland-adaptative variation in Tibet barley landrace population of China
Source: Front Plant Sci. 2023 May 10;14:1189642. doi: 10.3389/fpls.2023.1189642 (PMC10206316; doi:10.3389/fpls.2023.1189642)
Supplement: Supplementary file 3 [file DataSheet_1.docx]

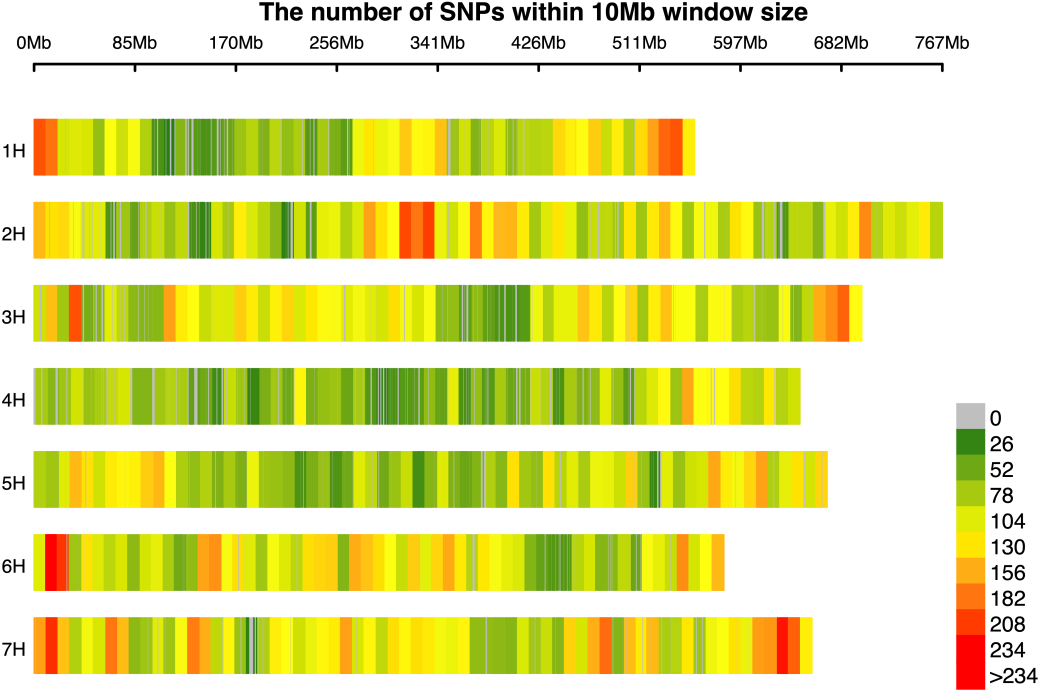


**Supplemental Figure 1. Heat map of SNPs number distribution on 7 barley chromosomes**

Coordinate axis at the top of the figure represents the chromosome length; rectangular segments along the chromosomes represent 10 Mb segments, gray represents regions with no SNPs; dark green, low numbers; increasing redness indicates increasing numbers.


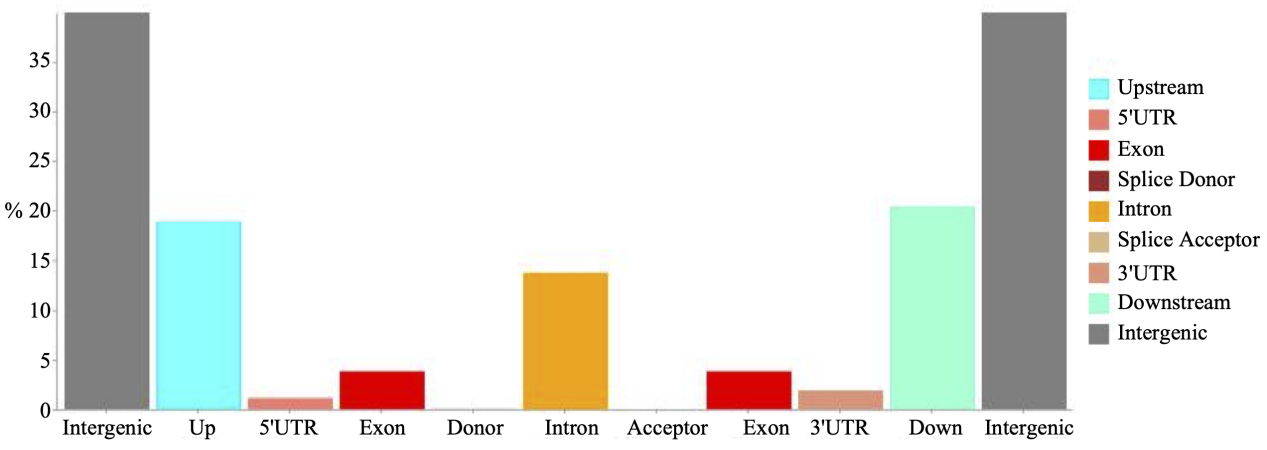


**Supplemental Figure 2. Histogram of numbers of SNPs in different genome structures**

Intergenic, Up, Down and Exon appear twice and showing duplicated value.

**Supplemental Figure 3. Population structure of 1,366 barley accessions, *K*=2 to 6**

Structure analysis showed that the inland barley group (IL) and Tibetan group (T) were significantly separated when *K* = 2. Tibetan accessions were divided into western Tibet (WT) and eastern Tibet (ET) types when *K* = 3. WT was divided into WT1 (high altitude) and WT2 (central Tibet), the ET groups remained basically unchanged when *K* = 4. In *K* = 5, WT2 was further divided into WT2 and WT3, whereas IL, WT1 and ET remained unchanged. *K* = 6 was most informative, where all accessions were divided into six groups, ET1, ET2, WT1, WT2, WT3 and IL


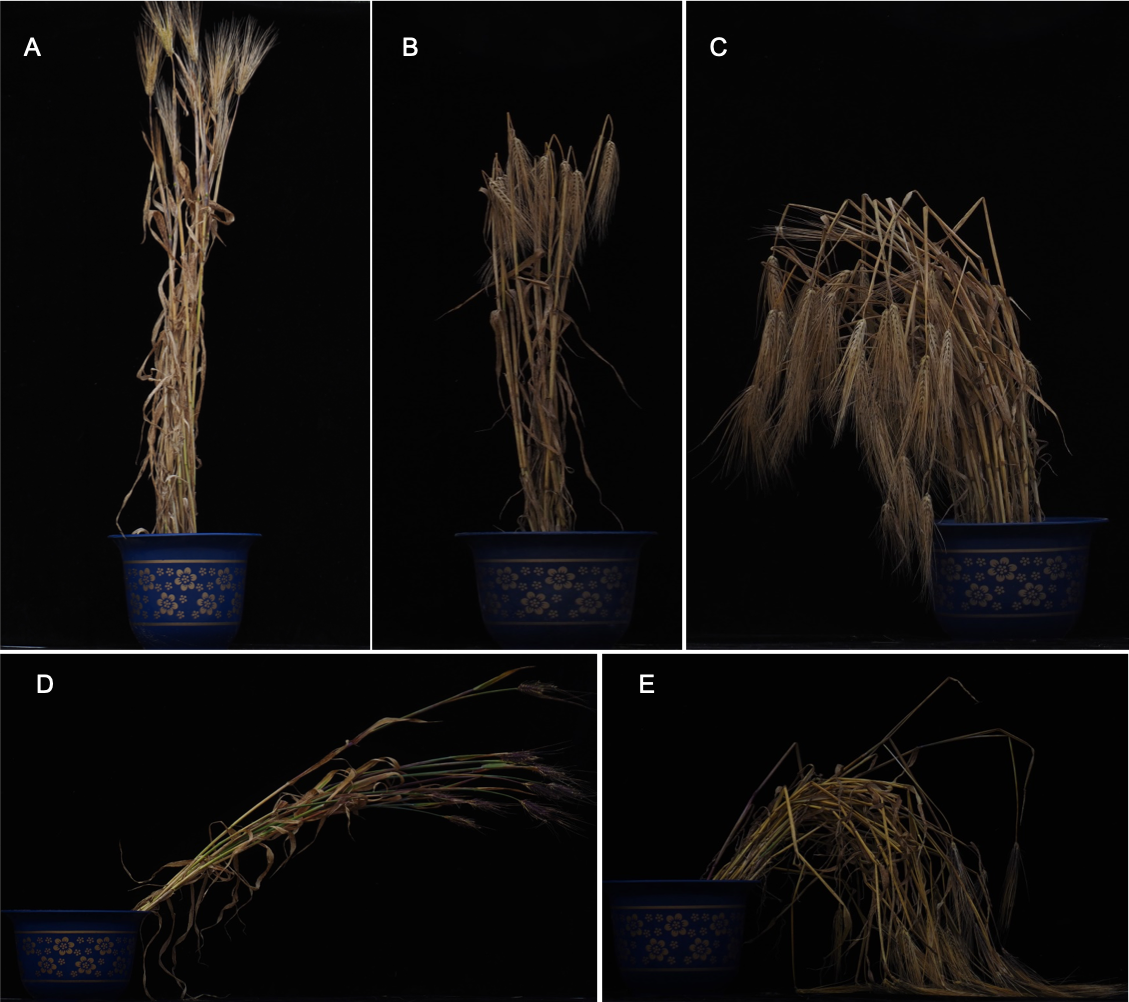


**Supplemental Figure 4. Lodging type of Qingke**

1. Erect: No Lodging.
2. Necking: Buckling of the peduncle below the ear.
3. Stem lodging is caused by one of the bottom two internodes buckling and resulting in the upper stem and ear lying horizontally.
4. Root lodging; permanent displacement of the cereal stems without any observable stem buckling.
5. Mixed: root and stem lodging.


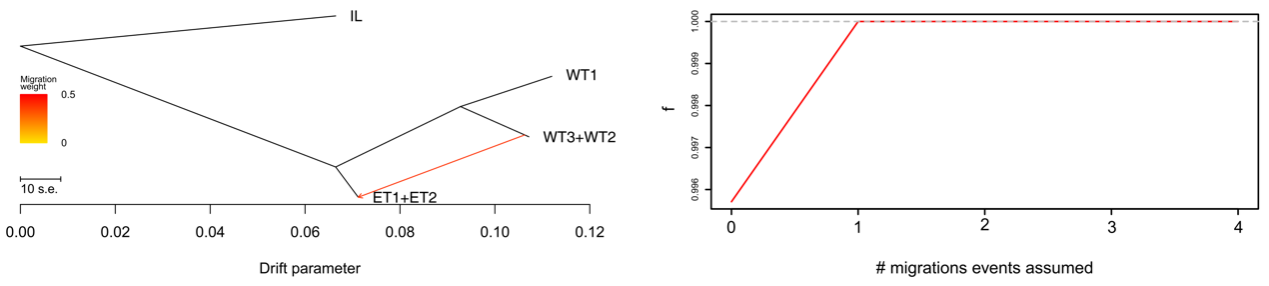


**Supplemental Figure 5. A single migration event among between WT23 and ET**


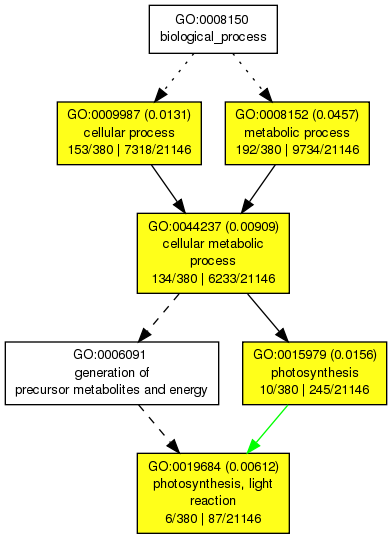


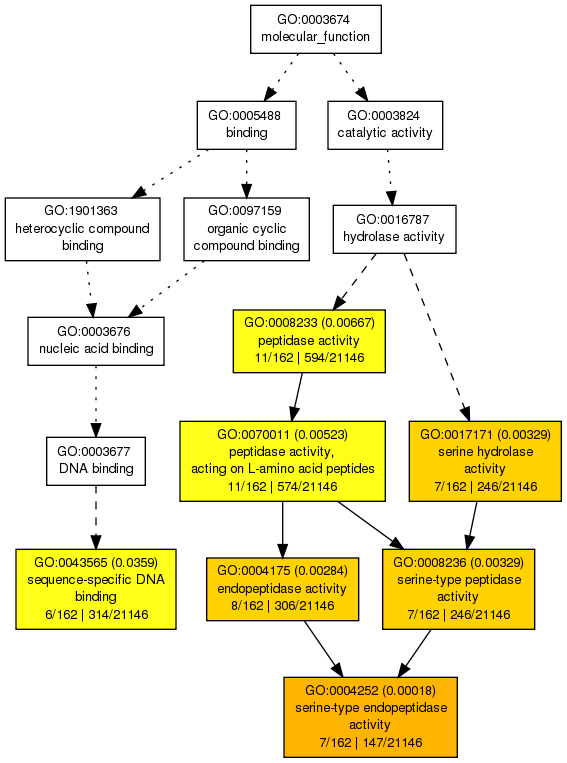


**Supplemental Figure 6. Significantly enriched genes in the differentiated chromosome 2H region in Qingke**


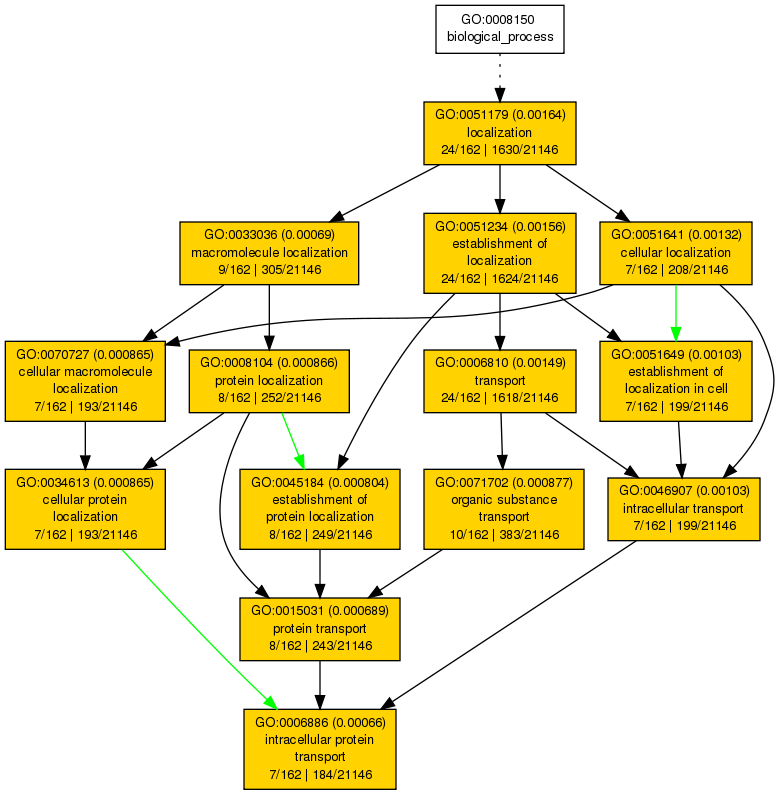


**Supplemental Figure 7. Significantly enriched genes in the differentiated chromosome 3H region in Qingke**


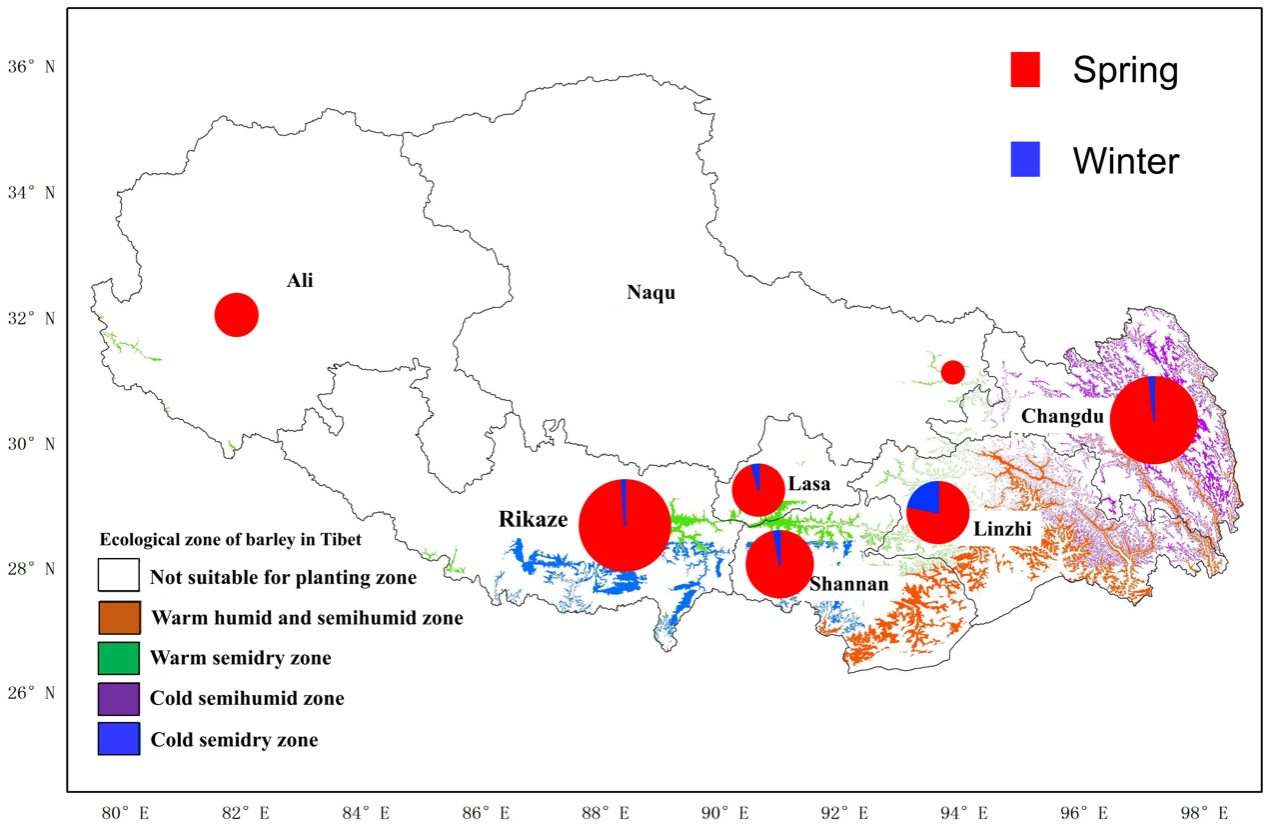


**Supplemental Figure 8. Geographical distribution of Qingke landraces with different growth habit**

**
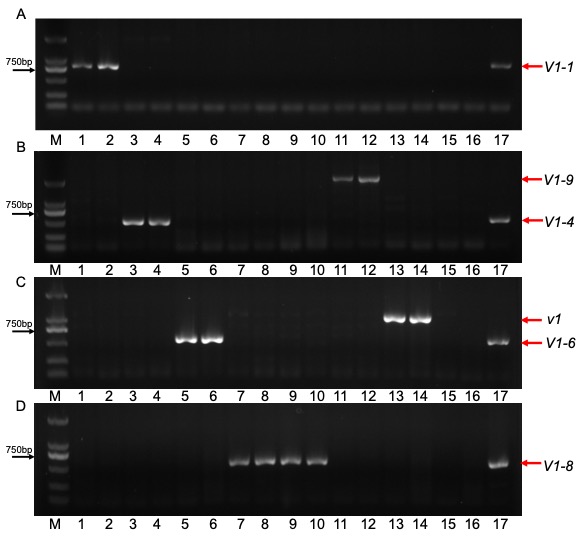
**

**Supplemental Figure 9. Haplotypes of *HvVRN1* in Qingke landraces**

M. DL2000 DNA ladder; 1. ZYM01082（*V1-1*）; 2. ZDM05444（*V1-1*）; 3. ZYM00011（*V1-4*）; 4. ZYM00020（*V1-4*）; 5. ZDM06601（*V1-6*）; 6. ZDM05008（*V1-6*）; 7. ZDM04214（*V1-8*）; 8. ZDM05119（*V1-8*）; 9. ZDM06599（*V1-8*）; 10. ZDM07210（*V1-8*）; 11. ZDM05536（*V1-9*）; 12. ZDM06508（*V1-9*）; 13. ZDM04900（*v1*）; 14. ZDM05170（*v1*）; 15. H2O; 16. Negative control; 17. Positive control.


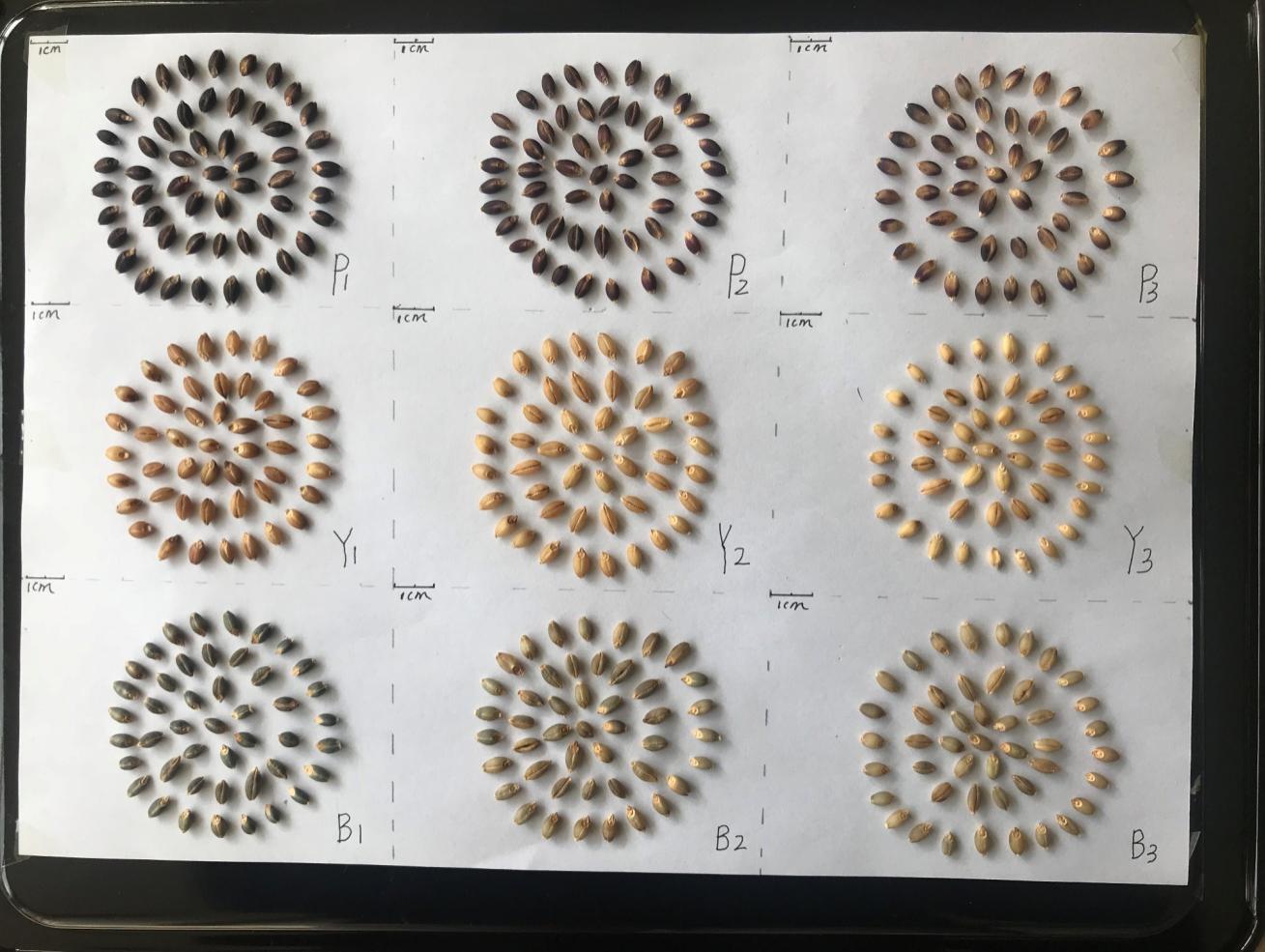


**Supplemental Figure 10. Color of Qingke Grains**

Seeds from a single plant were sun-dried and classified according to the color, white, blue or purple.


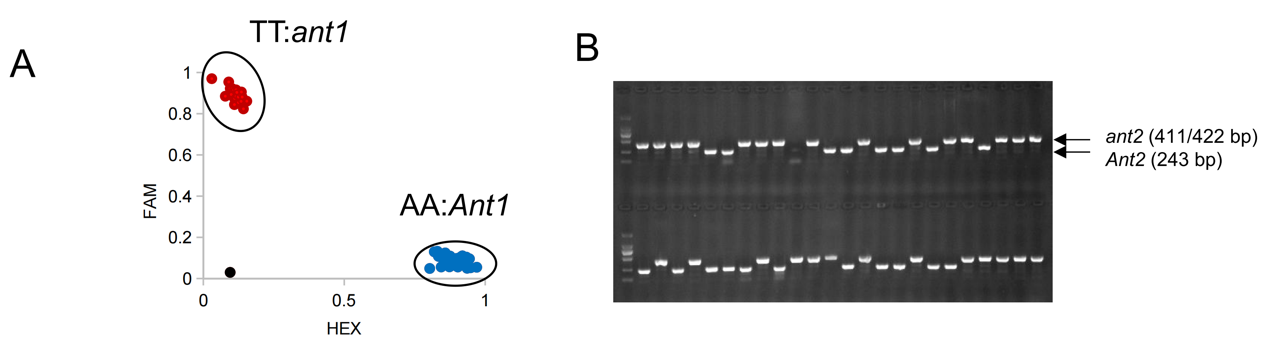


**Supplemental Figure 11. Molecular marker analysis of *HvAnt1* and *HVAnt2***

A. Cluster plot for the KASP genotyping assay of the key SNP variant site of *HvAnt1*. Blue and red represent the two distinct alleles, while black represents no DNA template negative controls. B. Agarose gel electrophoresis analysis of of the amplified PCR fragments for the 179/168 bp InDel of *HvAnt2* promoter. The upper and lower bands responsents the two types of *HvAnt2* alleles.


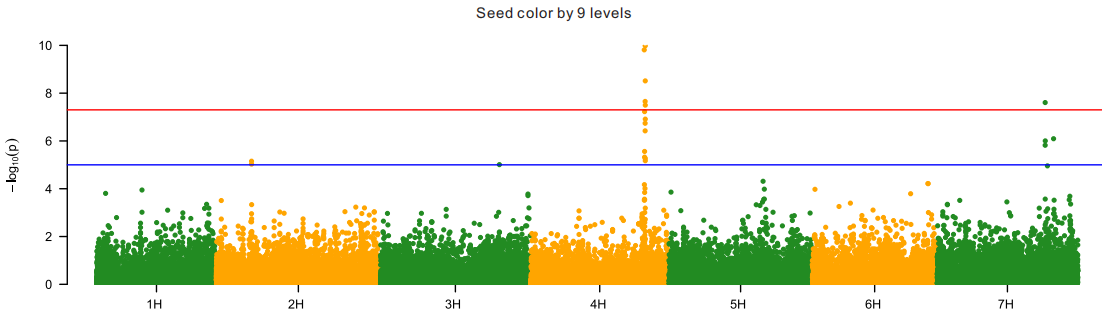


*Blx2*

MbhF35 cluster

**Supplemental Figure 12. GWAS analysis based on 9 colors in Qingke landraces**

Manhattan plots for GWAS analysis of blue grain. The red and blue horizontal lines denote suggestive threshold value *P* <5×10^-8^ and *P*<1×10^-5^, respectively.


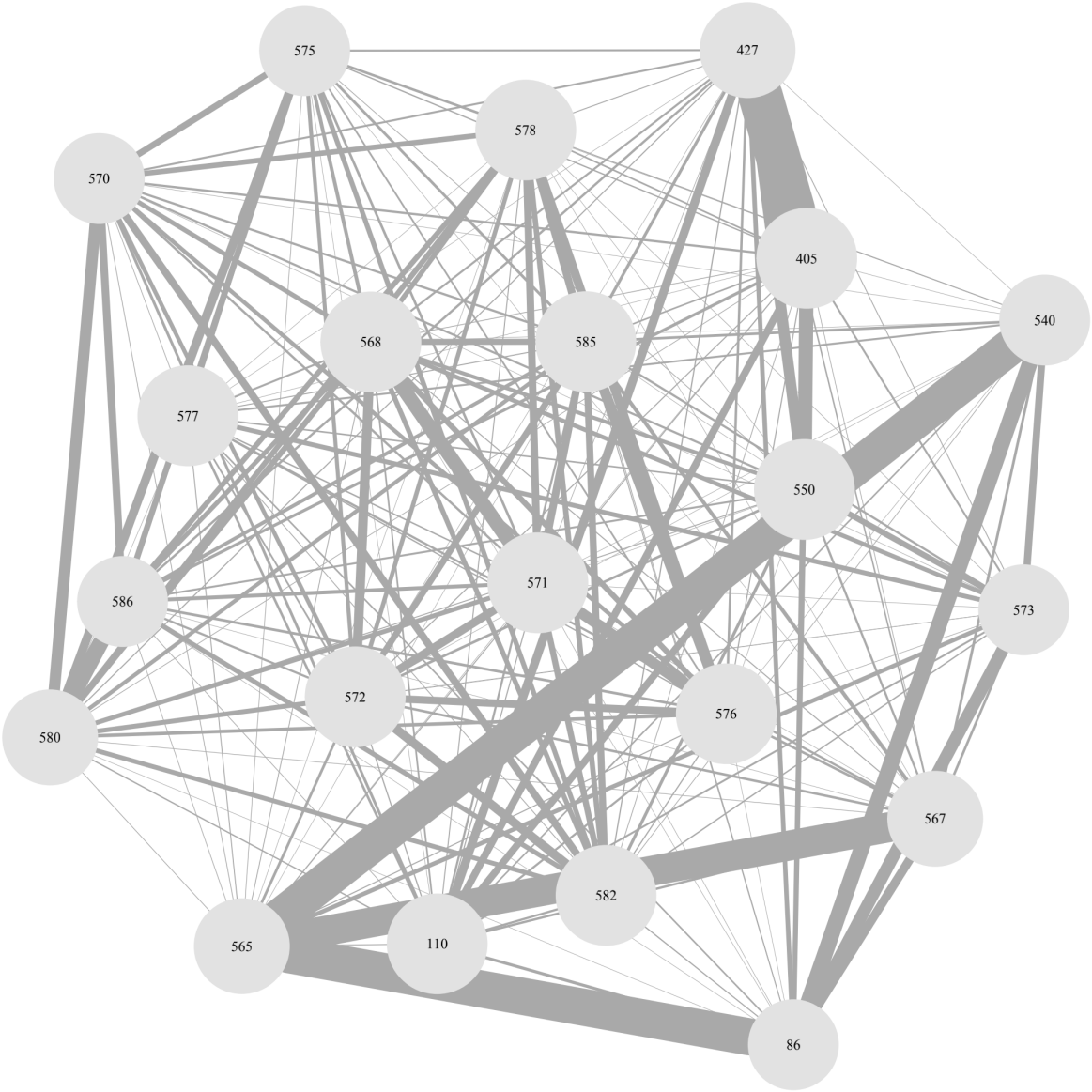


**Supplemental Figure 13. IBD (identity by descent) analysis among the 20 IL barley accessions**

Genetic exchange among 20 IL accessions. Accession “Suofu (565)” has highest similarity with others among which four were named Qingke (six-rowed and naked barley) such as “Ziqingke (86), Yijiqingke (427), Liujiaoqingke (550)” and “Duomangqingke (540)”. Names for other accessions are listed in Table S1.
